# A role for actin flexibility in thin filament-mediated contractile regulation and myopathy

**DOI:** 10.1038/s41467-020-15922-5

**Published:** 2020-05-15

**Authors:** Meera C. Viswanathan, William Schmidt, Peter Franz, Michael J. Rynkiewicz, Christopher S. Newhard, Aditi Madan, William Lehman, Douglas M. Swank, Matthias Preller, Anthony Cammarato

**Affiliations:** 1 Department of Medicine, Division of Cardiology, Johns Hopkins University, 720 Rutland Avenue, Baltimore, MD 21205 USA; 2 Institute for Biophysical Chemistry, Hannover Medical School, Carl-Neuberg-Straße 1, 30625 Hannover, Germany; 3 Department of Physiology and Biophysics, Boston University School of Medicine, 700 Albany Street St, Boston, MA 02118 USA; 4 Department of Biological Sciences and Center for Biotechnology and Interdisciplinary Studies, Rensselaer Polytechnic Institute, 110 8th Street, Troy, NY 12180-3590 USA; 5 Department of Physiology, Johns Hopkins University School of Medicine, 720 Rutland Avenue, Baltimore, MD 21205 USA

**Keywords:** Biochemistry, Biophysics, Cell biology, Computational biology and bioinformatics, Physiology

## Abstract

Striated muscle contraction is regulated by the translocation of troponin-tropomyosin strands over the thin filament surface. Relaxation relies partly on highly-favorable, conformation-dependent electrostatic contacts between actin and tropomyosin, which position tropomyosin such that it impedes actomyosin associations. Impaired relaxation and hypercontractile properties are hallmarks of various muscle disorders. The α-cardiac actin M305L hypertrophic cardiomyopathy-causing mutation lies near residues that help confine tropomyosin to an inhibitory position along thin filaments. Here, we investigate M305L actin in vivo, in vitro, and in silico to resolve emergent pathological properties and disease mechanisms. Our data suggest the mutation reduces actin flexibility and distorts the actin-tropomyosin electrostatic energy landscape that, in muscle, result in aberrant contractile inhibition and excessive force. Thus, actin flexibility may be required to establish and maintain interfacial contacts with tropomyosin as well as facilitate its movement over distinct actin surface features and is, therefore, likely necessary for proper regulation of contraction.

## Abstract (web summary)

The α-cardiac actin M305L hypertrophic cardiomyopathy-causing mutation is located near residues that help confine tropomyosin to an inhibitory position along thin filaments. Here the authors assessed M305L actin in vivo, in vitro, and in silico to characterize emergent pathological properties and define the mechanistic basis of disease.

## Introduction

Proper regulation of myocardial contraction and relaxation is essential for the heart to effectively pump blood, and even the most subtle disruptions in either process can elicit disease. Hypertrophic cardiomyopathy (HCM) is a heterogeneous heart disorder, with a prevalence estimated at 1 in 200, that is characterized by asymmetric myocardial growth, thickening of the interventricular septum, hyperdynamic contractile properties during systole, and impaired relaxation during diastole^1–3^. More than 1500 mutations in at least 11 genes are associated with HCM, which frequently engender a gain-in-sarcomeric-function^1,2,4–6^. Since the initial identification of an α-cardiac actin (*ACTC*) HCM-causing mutation in 1999, at least 12 additional ACTC variants have been reported in patients with HCM. The loci of the mutations are broadly distributed over the molecule with the M305L substitution located in subdomain 3 (SD3), close to the nucleotide-binding site (Supplementary Fig. 1A-C)^7^. Originally discovered in 2004, studies of M305L actin have focused predominantly on isolated protein measurements of mutation-induced changes in stability, polymerization properties, and nucleotide/phosphate release^8–11^. Much of the data are inconsistent, and in some cases contradictory, which confounds our understanding of the pathological basis of disease. Therefore, extending these analyses, to determine the mutation’s effects in situ and/or in vivo, may help resolve how M305L actin elicits HCM and, importantly, highlight universal properties of muscle biology. To date, such studies are lacking due to an inability to express M305L actin in cultured cardiomyocytes^10^ and the absence of animal models.

Cardiac muscle contraction is driven by cyclic interactions between myosin S1 heads of thick filaments and actin-containing thin filaments. It is regulated by troponin–tropomyosin (Tn–Tpm) complexes that, at rest, occlude myosin binding sites along filamentous actin (F-actin) and limit S1-thin filament binding^12,13^. Specifically, when sarcoplasmic Ca^2+^ is low, Tn binds to F-actin and Tpm and constrains Tpm to the B-state where it blocks and restricts myosin attachment. When elevated, Ca^2+^ binds to Tn, prompting the azimuthal movement of Tpm to the C-state that partially uncovers myosin binding sites. This facilitates weak-to-strong S1 cross-bridge binding and further displacement of Tpm to the M-state, which promotes additional S1 binding and contraction. During relaxation, following myosin detachment from F-actin, recent data suggest Tpm spontaneously moves back toward the energetically favorable, inhibitory B-state configuration^14^. Thus, Tpm must seamlessly translocate back-and-forth over the surface of F-actin, including any topological amino acid protrusions, to effectively regulate S1-thin filament cycling and, thereby, permit or prevent the production of force.

Tpm is an alpha-helical, coiled-coil dimer that binds seven adjacent actin monomers^15–17^. The dimers self-polymerize to produce a continuous strand that wraps around the F-actin helix. Locally, individual dimers associate weakly with the thin filament; yet, the full-length Tpm cable *as a whole* interacts strongly with F-actin^18–21^. This purported Gestalt-binding behavior of Tpm permits high affinity global binding that prevents detachment of the cable from F-actin, while low affinity local binding allows for the dynamic, azimuthal movements that are imperative for Tpm’s regulatory role in striated muscle^21,22^. Gestalt-binding relies on high complementarity between the three-dimensional, preshaped contours of Tpm and F-actin^19,21,23^. The structural complementarity permits the formation of roughly 30 interfacial electrostatic contacts that were predicted to establish the aforementioned weak, local F-actin–Tpm interactions and a broad energy basin, which biases Tpm to a location that limits S1–F-actin binding^24^. This energetically stable F-actin–Tpm conformation, known as the A-state, places Tpm in a location that is effectively the same as its B-state position on thin filaments replete with Tn; however, when not pinned down by Ca^2+^-free Tn, Tpm has greater azimuthal freedom^21^. Several actin residues, including K326, K328, and E334, are located along a continuous stretch of amino acids of SD3 and associate extensively with Tpm to help establish the A-state^24–28^. Notably, in vitro and in vivo findings illustrate that these associations facilitate Tn–Tpm-mediated steric blocking of S1 and, therefore, contribute to proper relaxation of cardiac and skeletal muscle^22,29–31^.

Here, we present a study that comprehensively assesses the consequences of M305L actin on striated muscle at the tissue, cellular, and molecular levels. Given the limited and often paradoxical findings regarding the intrinsic properties of M305L actin, we hypothesize that the lesion’s effects would be maximally manifested in higher order contractile systems replete with regulatory components and, ultimately, the mutation disrupts Tpm positioning and actomyosin inhibition. We show that in the *Drosophila melanogaster* heart, M305L mutant actin incorporates uniformly down cardiac thin filaments, increases periods of systolic tension generation, and impairs relaxation in a Ca^2+^-independent manner. Elevated expression of the variant in the indirect flight muscles (IFMs) has dose-dependent effects, progressively impairing flight and inducing destructive hypercontraction due to excessive, myosin-dependent force generation. Sinusoidal analysis of mutant IFM fibers reveals increased Ca^2+^ sensitivity of power development, while in vitro motility experiments indicate disinhibited M305L actin-Tpm filaments. Molecular dynamics (MD) simulations suggest that the flexibility and interconnectivity of M305L actin subregions are drastically reduced. Importantly, the amino acid stretch containing K326, K328, E334, and a residue that protrudes out from the F-actin backbone, P333, likewise displays substantially restricted motion and aberrant intramolecular communication. This could disrupt the formation of electrostatic interfacial contacts and the azimuthal stability of inhibitory Tpm positioning, as well as the unimpeded translocation of Tpm over the thin filament surface. Finally, computational chemistry predicts a poorly maintained A-state of M305L actin-Tpm filaments as reflected by a diminution of the energy basin that biases Tpm to an inhibitory configuration. In vivo, this lack of inhibitory bias likely impacts the B-state and accounts for the impaired regulatory behavior of Tn-containing thin filaments. Thus, our models recapitulate the earliest signs of HCM, including impaired relaxation and hyperdynamic contractile properties^3^. Furthermore, our findings are indicative of a gain-in-sarcomeric-function that results from a reduction in actin flexibility and concurrent destabilization of Tpm positioning along, and impaired movement over, thin filaments, which in humans may trigger HCM remodeling events.

## Results

### M305L actin induces cardiomyopathy in *Drosophila*

**Fig. 1:**
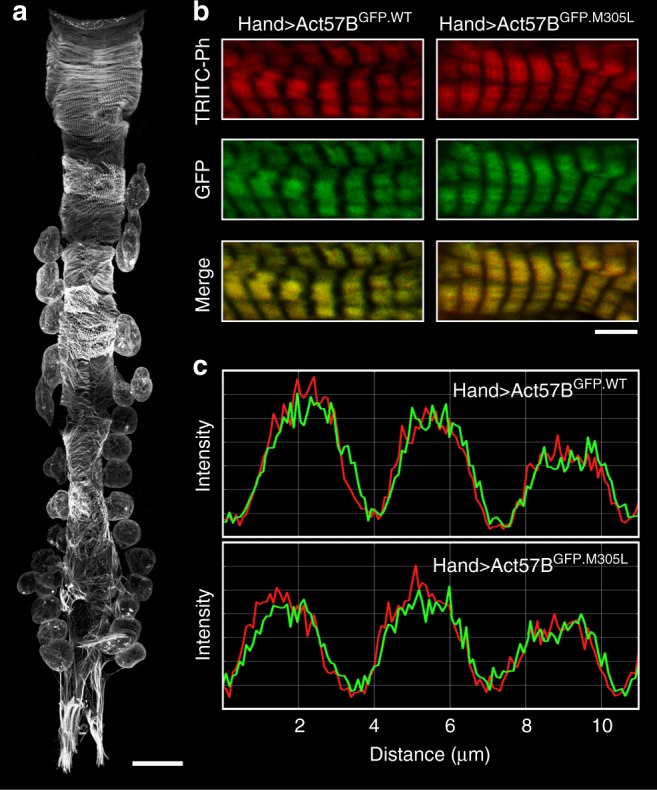
M305L actin incorporates evenly along *Drosophila* cardiac thin filaments. **a** Composite confocal image of a full-length *Hand* > *Act57B*^*GFP.WT*^ adult *Drosophila* heart tube. Four micrographs were acquired at ×40 magnification and computationally stitched together. Scale bar = 100 µm. **b** Fluorescent signals from Act57B^GFP.WT^ and Act57B^GFP.M305L^ actin, within fly cardiomyocytes, acquired at ×100 magnification. The GFP signals co-localized repetitively with TRITC-phalloidin (TRITC-Ph) signals. TRITC-Ph labels both transgenic and endogenous cardiac actin. Scale bar = 5 µm. **c** Fluorescent intensity line scans of sarcomeres in (**b**) reveal overlapping and congruent TRITC-ph (red) and GFP (green) signals, indicating that Act57B^GFP.M305L^ mutant actin copolymerized with endogenous cardiac actin along the length of the thin filaments, similar to control Act57B^GFP.WT^ transgenic actin.

Flies, like vertebrates, express specific actin isoforms in different muscles (Supplementary Fig. 2). *Act57B* is one of two sarcomeric actin genes expressed in the adult fly heart, while *Act88F* encodes all sarcomeric actin in the IFM^32–34^. The *Drosophila* heart consists of a single layer of bilateral rows of cardiomyocytes that form a simple linear tube (Fig. 1a)^35^. To determine if ectopically expressed M305L actin properly folds and incorporates evenly along *Drosophila* cardiac thin filaments, *Hand*^*4.2*^*-Gal4*-expressing virgin female flies were crossed with males carrying a transgene comprised of an upstream activating sequence (UAS) followed by GFP-labeled *Act57B*. The progeny (abbr. *Hand* > *Act57B*^*GFP.WT*^ and *Hand* > *Act57B*^*GFP.M305L*^) inherit both genes and express GFP-actin exclusively in the heart (Fig. 1a, b). At high (×100) magnification, we observed Act57B^GFP.M305L^ actin incorporating and extending uniformly along the entire thin filament in a manner that was indistinguishable from Act57B^GFP.WT^ actin (Fig. 1b). The GFP signals co-localized with TRITC-phalloidin signals, indicating homogenous co-polymerization of endogenous and transgenic actin isoforms (Fig. 1c). Act57B^GFP.M305L^ did not degrade or aggregate, nor was it excluded from the sarcomere. Importantly, quantitative western blot analysis confirmed that *Hand*^*4.2*^*-Gal4* drove comparable amounts of wildtype and mutant transgenic actin expression, with each comprising roughly 10% of total cardiac actin, in the respective fly hearts (Supplementary Fig. 3A).

**Fig. 2:**
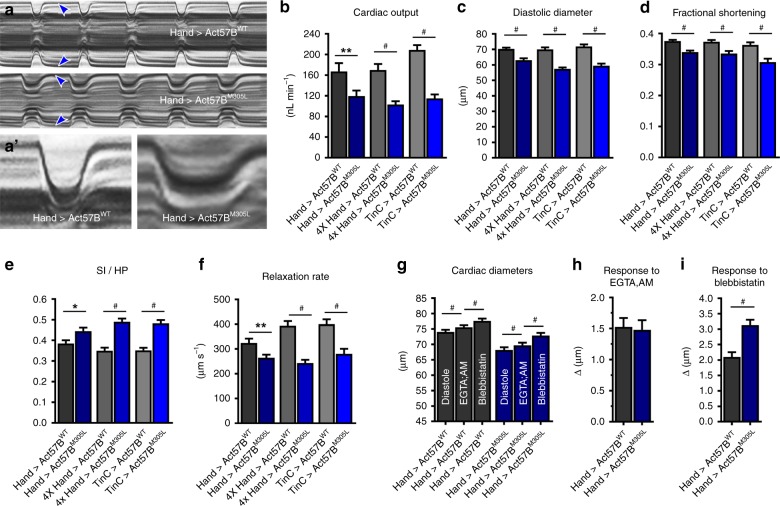
M305L actin triggers restrictive cardiac physiology and impairs relaxation. **a** M-mode kymograms generated from high-speed videos of beating, three-week-old *Hand* > *Act57B*^*WT*^ and *Hand* > *Act57B*^*M305L*^ hearts. These traces illustrate cardiac cycle dynamics and heart wall motion over time. Blue arrowheads demarcate the edges of the heart wall during diastole. Note the restricted diastolic diameter across the mutant cardiac tube. **a′** Individual systolic intervals taken from the traces shown in (**a**). Relative to *Hand* > *Act57B*^*WT*^, *Act57B*^*M305L*^*-*expressing cardiac tubes exhibited prolonged periods of tension generation, diminished shortening, and slower relaxation rates. **b**–**f** Heart-restricted expression of *Act57B*^*M305L*^ mutant actin significantly altered several indices of cardiac function, irrespective of genetic background, relative to the expression of *Act57B*^*WT*^. *Hand* > *, 4XHand* > , and *TinC* > *Act57B*^*M305L*^ *Drosophila* displayed decreased cardiac output, diastolic diameters, fractional shortening, and relaxation rates in addition to extended systolic periods relative to controls. SI/HP is the systolic interval over the time required for a complete cardiac cycle (i.e. diastolic plus systolic intervals). Significant differences between genotypes were determined using unpaired two-tailed *t*-tests (*n* = 31–45). *P ≤ 0.05, **P ≤ 0.01 and ^#^*P* ≤ 0.0001. **g** Significant, incremental increases in cardiac diameters were observed in *Hand* > *Act57B*^*WT*^ and *Hand* > *Act57B*^*M305L*^ Dros*ophila* following extra- and intracellular Ca^2+^ chelation and, again, upon blebbistatin exposure. Increases in cardiac dimensions due to EGTA-EGTA,AM and to blebbistatin were evaluated using repeated measures ANOVAs followed by Tukey’s multiple comparison tests of the matched groups (*n* = 30–31). ^#^*P* ≤ 0.0001. **h** The change in cardiac diameter in response to EGTA-EGTA,AM was similar between *Hand* > *Act57B*^*WT*^ and *Hand* > *Act57B*^*M305L*^ flies as determined by two-tailed unpaired *t*-tests (*n* = 30–31). **i** Blebbistatin treatment resulted in a significantly greater degree of heart wall relaxation in *Hand* > *Act57B*^*M305L*^ hearts relative to *Hand* > *Act57B*^*WT*^ hearts. Two-tailed unpaired *t*-tests were used to distinguish significant differences in cardiac diameter changes between genotypes (*n* = 30-31). ^#^*P* ≤ 0.0001. All data are presented as mean ± SEM. Source data are provided as a Source Data file.

To decipher the consequences of the HCM actin mutation on *Drosophila* cardiac performance, we evaluated beating hearts of female flies expressing *UAS-Act57B*^*WT*^ or *UAS-Act57B*^*M305L*^ transgenes that lacked the N-terminal GFP moiety, which our lab has shown can confound results^31^. M-Mode kymograms, generated using high-speed video microscopy and semi-automated optical heartbeat analysis software^36,37^, suggested cardiac-restricted expression of the actin variant reduced heart tube diameters, prolonged systole, and reduced the rate of myocardial relaxation relative to control (Fig. 2a). We quantified these and additional indices of cardiac function and assessed potential genotype- and dose-dependent effects by employing multiple cardiac-specific *GAL4* driver lines and distinct experimental conditions (Fig. 2b–f; Supplementary Fig. 3B, C). The latter entailed altering the rearing temperature, which results in a range of expression levels of *UAS* responder transgenes^38^. Based on the use of a nuclear-specific UAS-GFP reporter, the *4XHand-* (at 25 °C) and *TinC-Gal4* (at 29 °C) drivers were expected to yield ~4-fold higher *UAS-Act57B*^*WT*^ or *UAS-Act57B*^*M305L*^ expression levels in cardiomyocytes relative to *Hand*^*4.2*^*-Gal4* (at 25 °C) (Supplementary Fig. 3B). Regardless of the driver or temperature, *UAS-Act57B*^*M305L*^ actin expression significantly diminished cardiac output relative to controls, which was primarily due to restricted diastolic and systolic diameters coupled with significantly decreased fractional shortening (Fig. 2b–d, Supplementary Table 1). Flies expressing *UAS-Act57B*^*M305L*^ exhibited a significantly higher SI/HP ratio, which represents the proportion of time spent during the cardiac cycle generating systolic tension, and significantly decreased relaxation rates (Fig. 2e, f). The relative, physiological differences between control and mutant hearts were not exacerbated when M305L actin was expressed at higher doses using the stronger *4XHand-Gal4* or *TinC-Gal4* (at 29 °C) drivers (Supplementary Fig. 3C). Thus, comparable to the functional changes associated with human HCM, M305L actin expression in the fly heart, irrespective of genetic background, resulted in hyperdynamic contractile properties and it impaired relaxation and the ability to reestablish resting diastolic volumes.

HCM is frequently characterized by altered Ca^2+^ handling and elevated diastolic Ca^2+^ levels, which promote actomyosin associations and impair relaxation. To determine the mechanism of restricted cardiac diameters and to rule out a potential contribution from disrupted Ca^2+^ homeostasis, we incubated beating *Hand* > *Act57B*^*WT*^ and *Hand* > *Act57B*^*M305L*^ hearts in a solution containing 10 mM EGTA and 100 µM EGTA,AM^31,39^. EGTA-EGTA,AM chelates both extra- and intracellular Ca^2+^ and arrests contraction. Relative to that established during diastole, the heart tubes experienced a slight, yet significant, ~2% increase in diameters upon EGTA-EGTA,AM exposure (Fig. 2g). Thus, small amounts of residual intracellular Ca^2+^ are likely present in the hearts’ of both genotypes during diastole that stimulate actomyosin interactions and produce slightly contracted cardiomyocytes. However, there was no difference in the average change in cardiac diameters between *Hand* > *Act57B*^*WT*^ (Δ = 1.53 ± 0.15 µm) and *Hand* > *Act57B*^*M305L*^ (Δ = 1.48 ± 0.15 µm) upon treatment (Fig. 2h). This suggests that a similar diastolic Ca^2+^ level exists among both genotypes, which contributes equally to resting tension, and the significantly restricted *Act57B*^*M305L*^-expressing cardiac tubes do not result from elevated resting Ca^2+^. Unaltered Ca^2+^ handling was also supported by qPCR analysis of mRNA abundance (Supplementary Fig 4), which revealed no significant differences in transcript levels of L-type Ca^2+^ channels, ryanodine receptors, SERCA, Na^+^/Ca^2+^ exchangers, or in IP_3_ receptors between the lines.

Blebbistatin inhibits myosin and its addition to EGTA-EGTA,AM-treated fly hearts has been shown to elicit further increases in cardiac dimensions^31,40,41^. We confirmed blebbistatin’s effect on *Hand* > *Act57B*^*WT*^ and *Hand* > *Act57B*^*M305L*^ heart diameters (Fig. 2g, i), which suggests that chelation of extra- and intracellular Ca^2+^ does not induce complete relaxation of *Drosophila* cardiomyocytes due to a small population of S1 cross-bridges that continue to bind thin filaments. To evaluate the relative proportion of thin filament-bound, Ca^2+^-independent, diastolic cross-bridges present in *Hand* > *Act57B*^*WT*^ vs. *Hand* > *Act57B*^*M305L*^ hearts, we compared the cardiac responses to blebbistatin treatment. The blebbistatin-induced increase in diameter for *Hand* > *Act57B*^*WT*^ heart tubes was ~2.7% (Δ = 2.10 ± 0.15 µm), while that for *Hand* > *Act57B*^*M305L*^ was ~4.3% (Δ = 3.13 ± 0.17 µm) (Fig. 2i). Blebbistatin had a significantly greater effect on *Hand* > *Act57B*^*M305L*^ hearts relative to controls. This result is consistent with diastolic dysfunction and restrictive physiology that is independent of resting Ca^2+^ levels. Thus, the phenotype of *Hand* > *Act57B*^*M305L*^ mutant hearts is likely caused by excessively disinhibited, Ca^2+^-independent cross-bridge-binding to poorly regulated thin filaments, which results in enhanced basal tension and incomplete relaxation during diastole.

### M305L actin impairs flight and promotes IFM hypercontraction

**Fig. 3:**
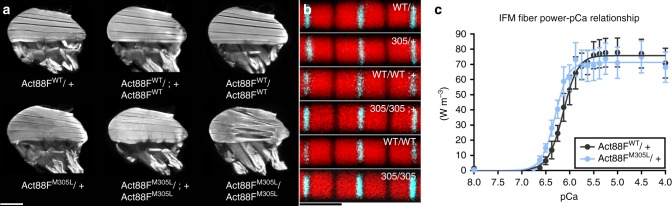
M305L actin causes IFM hypercontraction and enhances Ca^2+^ sensitivity. **a** Fluorescent micrographs of dorsal longitudinal IFMs (DLMs) of two-day-old *Act88F*^*WT*^ and *Act88F*^*M305L*^ *Drosophila*. *Act88F*^*WT*^/*+, Act88F*^*M305L*^/*+*, *Act88F*^*WT*^/*Act88F*^*WT*^;+, *Act88F*^*M305L*^/*Act88F*^*M305L*^;+ heterozygotes, and *Act88F*^*WT*^/*Act88F*^*WT*^ homozygotes displayed normal DLM morphology with the six fibers spanning the length of the thorax. *Act88F*^*M305L*^/*Act88F*^*M305L*^ homozygotes, however, demonstrated hypercontracted and torn DLMs. Scale bar = 250 µm. **b** Confocal images of consecutive sarcomeres along a single IFM myofibril from transgenic flies. Red, TRITC-phalloidin-labeled actin; Cyan, immunolabeled α-actinin. WT/+ = *Act88F*^*WT*^/*+*, 305/+ = *Act88F*^*M305L*^/*+*, WT/WT;+ = *Act88F*^*WT*^/*Act88F*^*WT*^;+, 305/305;+ = *Act88F*^*M305L*^/*Act88F*^*M305L*^;+, WT/WT = *Act88F*^*WT*^/*Act88F*^*WT*^, and 305/305 = *Act88F*^*M305L*^/*Act88F*^*M305L*^. IFM thin filament lengths did not significantly differ among the genotypes (Supplementary Fig. 5B) as determined by a Kruskal-Wallis one-way ANOVA with Dunn’s post hoc test (*n* = 200–211). Scale bar = 2.5 µm. **c** The power-pCa relationship of *Act88F*^*M305L*^/*+* IFM fibers revealed a significant leftward shift in Ca^2+^ sensitivity (see Table 2), indicating less Ca^2+^ is required for activation. Significance was assessed via an unpaired two-tailed *t*-test (*n* = 10). Data points are mean ± SEM and were fit by the Hill equation. Source data are provided as a Source Data file.

*Drosophila* IFMs are well suited for structural and mechanical analyses and are highly sensitive to sarcomeric perturbations, making them ideal for studying the phenotypic and functional effects of muscle protein mutations^31,39,42–46^. We created *Act88F*^*WT*^ and *Act88F*^*M305L*^ flies that expressed transgenic and endogenous actin at different ratios by backcrossing the transgene into a flightless, *Act88F*-null strain (see Materials and Methods). *Act88F*^*M305L*^/+ flies, which express one transgenic and one endogenous copy of actin, exhibited a significant decrease in flight ability compared to *Act88F*^*WT*^/+ controls (Table 1). Despite flight differences, the IFMs of both lines appeared normal, each with six undisturbed dorsal longitudinal muscle fibers (DLMs) spanning the length of the thorax (Fig. 3a). When the ratio of transgenic to endogenous actin was increased to two to one, the flight ability of both the wildtype and mutant lines was diminished; however, the impairment observed for *Act88F*^*M305L*^/*Act88F*^*M305L*^;+ was significantly greater than that for *Act88F*^*WT*^/*Act88F*^*WT*^;+ flies (Table 1). Nonetheless, the majority of *Act88F*^*M305L*^/*Act88F*^*M305L*^;+ *Drosophila* had intact IFMs with only a small percentage of flies displaying minor disturbances and breaks in their DLMs (Fig. 3a, Supplementary Fig. 5A). Homozygous expression of wildtype transgenic actin in *Act88F*^*WT*^/*Act88F*^*WT*^, in an endogenous IFM-actin null background, did not rescue the flightless phenotype associated with the *Act88F*-null strain (Table 1), potentially due to insufficient transgene expression^31^. *Act88F*^*WT*^/*Act88F*^*WT*^ IFM morphology, however, was phenotypically normal. *Act88F*^*M305L*^/*Act88F*^*M305L*^ *Drosophila* were similarly flightless (Table 1), yet their IFMs exhibited destructive hypercontraction with 100% penetrance (Fig. 3a). This phenotype can result from excessive and/or unregulated force generation due to Tpm-based disinhibition of actomyosin interactions^40,44^. Thus, unlike what was observed in the fly heart, the IFM showed progressively worse phenotypes with increased M305L actin expression.

**Table 1:** Flight indices of *Drosophila* expressing varying amounts of transgenic versus endogenous *Act88F*.

Genotype	Tg:Endo	Flight index	n
*Act88F*^*WT*^/+	1:1	5.05 ± 0.08	206
*Act88F*^*M305L*^/+	1:1	3.85 ± 0.11^#^	212
*Act88F*^*WT*^/*Act88F*^*WT*^;+	2:1	3.98 ± 0.11	199
*Act88F*^*M305L*^/*Act88F*^*M305L*^;+	2:1	1.74 ± 0.08^#^	227
*Act88F*^*WT*^/*Act88F*^*WT*^	2:0	0.00 ± 0.00	222
*Act88F*^*M305L*^/*Act88F*^*M305L*^	2:0	0.00 ± 0.00	222

Tg:Endo refers to the ratio of transgenic to endogenous *Act88F* gene copy numbers. Data are presented as mean ± SEM. ^#^Significantly different from the corresponding WT control line as determined by the Mann–Whitney test (*P* ≤ 0.0001). Source data are provided as a Source Data file.

Since the M305L mutation has been shown to reduce F-actin lengths in vitro^10^, and thin filament length is an important determinant of force production in vivo^47^, we determined whether a mutation-induced change in thin filament length might contribute to M305L-mediated IFM pathology. The average thin filament lengths, measured from confocal micrographs of TRITC-phalloidin labeled IFM myofibrils from two-day-old *Act88F*^*WT*^/+ (1.40 ± 0.01 µm), *Act88F*^*M305L*^/+ (1.38 ± 0.01 µm), *Act88F*^*WT*^/*Act88F*^*WT*^;+ (1.39 ± 0.01 µm), *Act88F*^*M305L*^/*Act88F*^*M305L*^;+ (1.37 ± 0.01 µm), *Act88F*^*WT*^/*Act88F*^*WT*^ (1.39 ± 0.01 µm), and *Act88F*^*M305L*^/*Act88F*^*M305L*^ (1.37 ± 0.01 µm) *Drosophila*, were not significantly different (Fig. 3b, Supplementary Fig. 5B). Thus, disrupted thin filament length is likely not a contributing factor to Act88F^M305L^-induced hypercontraction.

### M305L actin enhances Ca^2+^ sensitivity of IFM fibers

At the cellular and molecular levels, HCM-causing thin filament mutations are frequently associated with impaired contractile regulation as characterized by elevated Ca^2+^ sensitivity of activation^4,5^. To ascertain the influence of the M305L actin mutation on Ca^2+^ sensitivity and mechanical properties of muscle cells, we conducted sinusoidal analysis of skinned IFM fibers. *Drosophila* IFM isometric tension is exceedingly low, especially at minimal and intermediate Ca^2+^ concentrations, which greatly compromises signal to noise ratio^46^. Therefore, IFM power and elastic modulus are more amenable to producing an accurate measurement of Ca^2+^ sensitivity. We determined the power generated in IFMs as a function of Ca^2+^ from heterozygous *Act88F*^*WT*^*/+* and *Act88F*^*M305L*^*/+* flies. *Act88F*^*WT*^*/+* and *Act88F*^*M305L*^*/+* IFM fibers were analyzed since they appeared overtly intact and structurally normal (Fig. 3a) and to best represent the dominant condition observed in afflicted patients. Act88F^M305L^-containing fibers displayed increased Ca^2+^ sensitivity, as evidenced by a significant leftward shift in pCa_50_ compared to Act88F^WT^ controls with no difference in maximum power generated (Fig. 3c, Table 2). The slope of the curve (Hill coefficient) was not significantly different between fiber types indicating no change in cooperativity of thin filament activation. Elastic modulus was measured at 500 Hz and also revealed a leftward shift in pCa_50_ in mutant fibers with no change in Hill coefficient or maximum elastic modulus (Supplementary Fig 5C and Supplementary Table 2). The shift in Ca^2+^ sensitivity was likely not due to changes in cross-bridge cycling kinetics, as the frequency at which maximum power was generated (*f*_*max*_) was not significantly different between Act88F^M305L^ and Act88F^WT^ actin-containing fibers (Table 2). Similarly, muscle apparent rate constants 2πb, primarily influenced by steps associated with myosin binding to actin, the power stroke and Pi release, and 2πc, largely defined by the strongly bound steps including ADP release and ATP-induced myosin detachment from actin, were not significantly affected by the mutation.

**Table 2:** Power measurements and muscle apparent rate constants determined from IFM mechanical analysis.

Genotype	pCa_50_	Hill coefficient	Max power (W m^−3^)	*f*_max_ (Hz)	*2*πb (s^−1^)	*2*πc (s^−1^)	*n*
*Act88F*^*WT*^/+	6.11 ± 0.05	3.52 ± 0.3	77.7 ± 9.5	188.5 ± 3.9	1702 ± 64	2697 ± 69	10
*Act88F*^*M305L*^/+	6.30 ± 0.03**	3.94 ± 0.6	69.9 ± 10.3	191.0 ± 3.8	1724 ± 50	2785 ± 136	10

Data are presented as mean ± SEM. ^**^Significantly different from control *Act88F*^*WT*^/+ fibers as determined by Student’s *t* test (*P* ≤ 0.01). Source data are provided as a Source Data file.

### M305L cardiac actin induces myosin-dependent IFM pathology

**Fig. 4:**
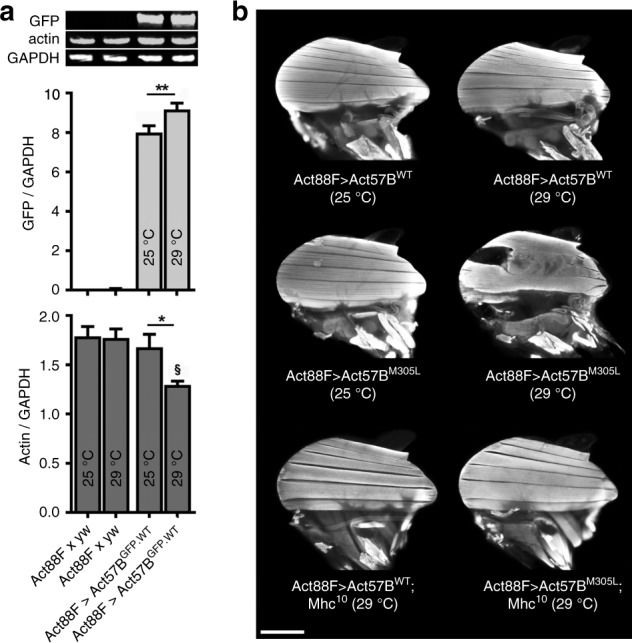
High dose overexpression of M305L cardiac actin disrupts the IFM in a myosin-dependent fashion. **a** Quantitative western blot analysis of Act57B^GFP.WT^ and endogenous actin was performed on IFMs from the progeny of *Act88F* x *yw* (control) and *Act88F* > *Act57B*^*GFP.WT*^ *Drosophila* raised at 25 °C and 29 °C, two days after eclosion. Representative western blot, probed with antibodies that targeted GFP, actin, and GAPDH, showing expression of Act57B^GFP.WT^ actin in *Act88F* > *Act57B*^*GFP.WT*^ flies and an absence of GFP-actin in control IFMs. The GFP-actin intensities (normalized to GAPDH) were significantly higher in flies raised at 29 °C. Actin intensities (normalized to GAPDH) revealed that *Act88F* > *Act57B*^*GFP.WT*^ *Drosophila* raised at 29 °C had a significant reduction in non-tagged, endogenous IFM actin. Quantification was performed on six independent biological replicates with three technical replicates each. Significance was assessed via one-way ANOVA with Tukey’s multiple comparison test. *P ≤ 0.05, **P ≤ 0.01; §*P* ≤ 0.01 relative to *Act88F* x *yw* at 25 °C or 29 °C. All data are presented as mean ± SEM. Source data are provided as a Source Data file. **b** Fluorescent micrographs of dorsal longitudinal IFMs (DLMs) of two-day-old *Act88F* > *Act57B*^*WT*^ and *Act88F* > *Act57B*^*M305L*^ *Drosophila. Act88F* > *Act57B*^*M305L*^ flies raised at 25 °C displayed similar IFM morphology to *Act88F* > *Act57B*^*WT*^ flies. Conversely, *Act88F* > *Act57B*^*M305L*^ flies raised at 29 °C, with elevated mutant actin, showed hypercontracted IFMs, with the middle fibers pulling away from anterior attachment sites. A reduction in IFM myosin content, due to the presence of a single copy of the *Mhc*^*10*^ (IFM-specific MHC null) allele, had no effect on the gross DLM morphology of *Act88F* > *Act57B*^*WT*^;*Mhc*^*10*^/+ *Drosophila* (raised at 29 °C)*. Act88F* > *Act57B*^*M305L*^;*Mhc*^*10*^/+ *Drosophila* (raised at 29 °C) displayed a complete rescue of the hypercontracted phenotype. Scale bar = 250 µm.

To determine whether misexpression of M305L cardiac actin in skeletal muscle would also elicit a hypercontractile phenotype, and if this potentially was myosin-dependent, *UAS-Act57B*^*WT*^ and *UAS-Act57B*^*M305L*^ transgenes were expressed at variable doses with the *Act88F-Gal4* IFM driver. The flies were raised at ambient (25 °C) or elevated (29 °C) temperature to increase protein load. The effect of elevated temperature on transgenic actin expression was confirmed by crossing *Act88F-Gal4* females with *UAS-Act57B*^*GFP.WT*^ males; control flies were the progeny of *Act88F-Gal4* females crossed with *yw* males. IFMs from *UAS-Act57B*^*GFP.WT*^-driven and control flies, reared at 25 °C or 29 °C, were dissected and subjected to quantitative western blot analysis using actin and GFP antibodies (Fig. 4a). GFP and actin signal intensities were measured and normalized to that from GAPDH. As expected, GFP was undetected in controls maintained at either temperature. IFMs from *Act88F* > *Act57B*^*GFP.WT*^ flies, however, had substantial amounts of GFP-actin, with elevated temperature inducing significantly higher expression relative to that at 25 °C. Transgenic cardiac GFP-actin comprised ~20% and ~30% of total IFM actin in flies maintained at 25 °C and 29 °C, respectively (Supplementary Fig. 5D). Endogenous actin abundance was unaffected when *Act88F* > *UAS-Act57B*^*GFP.WT*^ flies were raised at 25 °C. Notably, increased expression of *Act57B*^*GFP.WT*^ coincided with a significant reduction in the amount of endogenous actin (Fig. 4a). This potentially indicates that there is a maximum level for actin overexpression in the IFM, beyond which stoichiometry is disrupted and compensatory downregulation of the endogenous isoform ensues.

Since GFP-labeled actin can induce flightlessness^30,48^, flight tests were performed on two-day-old *Act88F* > *Act57B*^*WT*^ and *>Act57B*^*M305L*^ *Drosophila*. Low dose ectopic expression (25 °C) of cardiac *UAS-Act57B*^*M305L*^ actin had no effect on flight ability relative to expression of *UAS-Act57B*^*WT*^ (Table 3), and fluorescence microscopy revealed normal IFM morphology (Fig. 4b). However, higher expression levels (29 °C) of *UAS-Act57B*^*WT*^ and *UAS-Act57B*^*M305L*^ actin resulted in a reduction and a loss of flight, respectively (Table 3). *Act88F* > *Act57B*^*WT*^ *Drosophila* raised at 29 °C showed normal IFM structure while *Act88F* > *Act57B*^*M305L*^ had damaged DLMs, with the middle fibers bunching and pulling away from their anterior attachment sites, indicative of hypercontracted muscle (Fig. 4b). Thus, independent of the actin isoform, the M305L amino acid substitution induced dose-dependent hypercontraction of skeletal muscle that did not support function.

**Table 3:** Flight indices of *Act88F* > *Act57B*^*WT*^ and *Act88F* > *Act57B*^*M305L*^ flies at 25 °C and 29 °C.

Genotype	Rearing temp (°C)	Flight index	*n*
*Act88F* > *Act57B*^*WT*^	25	5.21 ± 0.08	316
*Act88F* > *Act57B*^*M305L*^	25	5.22 ± 0.07	408
*Act88F* > *Act57B*^*WT*^	29	2.74 ± 0.12	321
*Act88F* > *Act57B*^*M305L*^	29	0.31 ± 0.06^#^	204
*Act88F* > *Act57B*^*WT*^*; Mhc*^*10*^	29	0.00 ± 0.00	103
*Act88F* > *Act57B*^*M305L*^; *Mhc*^*10*^	29	0.00 ± 0.00	114

Data are presented as mean ± SEM. ^#^Significantly different from *Act88F*>*Act57B*^*WT*^ (29 °C) as determined by the Mann-Whitney test (*P* ≤ 0.0001). Source data are provided as a Source Data file.

To verify that the fiber tears and bunching in *Act88F* > *Act57B*^*M305L*^ flies reared at 29 °C were myosin dependent, *Act88F* > *Act57B*^*WT*^ and *Act88F* > *Act57B*^*M305L*^ females were crossed to *Mhc*^*10*^*/Mhc*^*10*^ IFM myosin-null homozygous males to create progeny that express *Act57B*^*WT*^ (*Act88F* > *Act57B*^*WT*^;*Mhc*^*10*^*/+*) or *Act57B*^*M305L*^ (*Act88F* > *Act57B*^*M305L*^;*Mhc*^*10*^*/+*) in combination with a 50% reduction in myosin. Strikingly, *Act88F* > *Act57B*^*M305L*^;*Mhc*^*10*^*/+ Drosophila* raised at 29 °C exhibited IFM morphology that was identical to that of *Act88F* > *Act57B*^*WT*^;*Mhc*^*10*^*/+* flies, with complete protection from M305L actin-induced hypercontraction (Fig. 4b). This demonstrates that excessive myosin-dependent force generation is the underlying cause for fiber auto-destruction in Act57B^M305L^-containing IFMs, and that the mutation plausibly disrupts the ability of the thin filament to properly regulate and oppose S1 cross-bridge binding.

### M305L actin reduces Tpm-based inhibition of F-actin motility

Our results, obtained using both cardiac and skeletal muscle, collectively imply that the ACTC variant impairs Tpm-based steric blocking of S1 binding. Therefore, we directly measured the ability for the M305L mutation to upset Tpm positioning, and thus the inhibitory A-state, in vitro by quantifying the percent of Act88F^WT^ vs. Act88F^M305L^ filaments that were propelled by varying concentrations of myosin. Previous studies have shown that under low myosin loading concentrations, the velocity and/or the percent of motile actin filaments were significantly reduced upon the addition of Tpm, consistent with an inhibitory F-actin–Tpm configuration^49–52^. Homogenous populations of IFM actin were purified from *Act88F*^*WT*^*/Act88F*^*WT*^ and *Act88F*^*M305L*^*/Act88F*^*M305L*^ flies, labeled with TRITC-phalloidin, and subjected to in vitro motility analysis. The M305L mutation had no effect on the percent of filaments that were propelled by myosin in the absence of Tpm (Table 4). Moreover, as found earlier^11^, both wildtype and mutant F-actin displayed similar sliding velocities (Supplementary Fig 6), which suggests the M305L mutation does not overtly influence actomyosin associations and the disease mechanism involves additional, higher order regulatory components. Consistent with this hypothesis, in the presence of vertebrate cardiac Tpm, the mutation elicited a significant increase in the percent of motile Act88F^M305L^ relative to Act88F^WT^ filaments at all myosin concentrations tested (Table 4). These results indicate that the M305L substitution likely destabilizes the native, inhibitory F-actin–Tpm A/B-state.

**Table 4:** Percent of motile wildtype and mutant actin–Tpm filaments determined from in vitro analysis.

Filament type	±Tpm	[Myosin] (µg ml^−1^)	Filaments moving (%)	*n*
F-Act88F^WT^	−	12.5	31.02 ± 1.64	22
F-Act88F^M305L^	−	12.5	32.57 ± 2.34	21
F-Act88F^WT^	+	12.5	14.96 ± 1.49	20
F-Act88F^M305L^	+	12.5	21.17 ± 2.11*	21
F-Act88F^WT^	−	50	68.11 ± 1.51	16
F-Act88F^M305L^	−	50	68.00 ± 2.30	20
F-Act88F^WT^	+	50	56.43 ± 1.04	21
F-Act88F^M305L^	+	50	67.81 ± 1.37^#^	21
F-Act88F^WT^	−	100	64.09 ± 1.64	19
F-Act88F^M305L^	−	100	67.89 ± 1.61	22
F-Act88F^WT^	+	100	62.87 ± 1.83	20
F-Act88F^M305L^	+	100	71.16 ± 1.78**	22

Data are presented as mean ± SEM. *P≤0.05, **P≤0.01, ^#^*P*≤0.0001 Significantly different from F-Act88F^WT^+Tpm. Significant differences between filament types ± Tpm at each myosin concentration were assessed using a two-way ANOVA with Bonferroni post-tests. Source data are provided as a Source Data file.

### M305L actin exhibits low flexibility during MD simulations

**Fig. 5:**
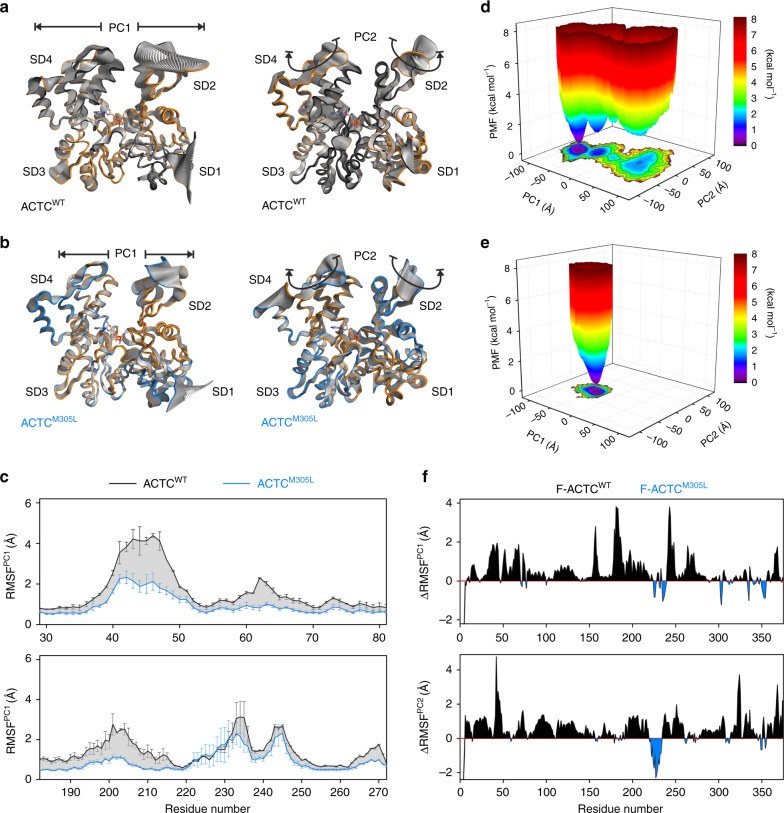
The M305L mutation decreases actin flexibility in MD simulations. **a** Projections of the largest structural fluctuations according to the first principal component (PC) (left panel) and the second PC (right panel) of a representative cMD simulation of an ACTC^WT^ monomer. The projections indicate major positional deviations of SD2 and SD4, which follow hinge domain (PC1) as well as rotational movements (PC2). **b** Projections of PC1 and PC2 of a representative ACTC^M305L^ monomer cMD simulation illustrate decreased protein motions compared to wildtype. **c** Root mean square fluctuations along PC1 of SD2 (upper panel) and SD4 (lower panel) calculated from monomer cMD simulations. Both SD2 and SD4 show markedly higher fluctuations in ACTC^WT^ vs. ACTC^M305L^. Data are represented as mean ± SEM (*n* = 3). **d** The free energy landscape of ACTC^WT^, calculated as the potential of mean force (PMF) according to the largest structural motions in the protein, as derived from PCA of two combined 500 ns enhanced sampling MD simulations. The color bar represents the PMF value in kcal mol^−1^. **e** The free energy landscape of ACTC^M305L^ shows a much narrower energy basin as compared to ACTC^WT^, indicating a highly populated state with reduced flexibility. Data were obtained from two combined 500 ns enhanced sampling MD simulations. The color bar represents the PMF value in kcal mol^−1^. **f** Root mean square fluctuations obtained from F-actin cMD simulations, given as the difference of ACTC^WT^−ACTC^M305L^, along PC1 (upper panel) and PC2 (lower panel) confirm that the major protomer structural fluctuations persist in the filamentous actin form, and they are decreased in mutant actin. Source data are provided as a Source Data file.

To investigate mutation-induced changes in structural properties and protein dynamics, which may provide insight into the mechanism of Tpm mispositioning as well as fundamental properties of thin filament biology, we performed MD simulations of wildtype and M305L human monomeric (G-actin) and F-actin structural models (Supplementary Table 3). First, triplicate, 200 ns classical MD (cMD) simulations were carried out to characterize the effects of the mutation on G-actin. Principal component analysis (PCA)^53^ was employed to reduce the dimensionality of the cMD trajectories and resolve dominant and concerted conformational changes in the protomers (Fig. 5a, b, Supplementary movies 1-4). Most of the conformational space sampled during cMD simulations was represented by only a few principal components (PCs), with the first three PCs comprising 70 to 80% of the variance in atomic positional fluctuations. The dominant motions in wildtype and mutant G-actins primarily included flexion and extension movements of SD2 and 4 (PC1) around a hinge joint localized in the interdomain connection between SD1 and 3, and a rotational twisting of the proteins (PC2). Projecting the two PCs onto the G-actin structural models revealed markedly reduced flexibility of ACTC^M305L^ relative to ACTC^WT^, as indicated by the decreased amplitude of structural rearrangements in the mutant, both along PC1 and PC2 (Fig. 5a, b). This was corroborated by decreased root-mean-square-deviations (RMSD) of ACTC^M305L^ subdomains, particularly of SD2 and SD4 (Supplementary Fig. 7A). Excluding the highly flexible DNase-binding loop (residues 39 to 50) and hydrophobic plug (residues 263 to 271), which were shown earlier to retain high mobility in monomeric and F-actin^54^, the backbone atoms of SD2 and SD4 of mutant G-actin exhibited RMSDs up to 3.2 Å and 3.3 Å respectively, over the cMD simulations, as compared to 5.6 Å and 4.9 Å of wildtype. Accordingly, SD2 and SD4 of ACTC^M305L^ remained closer together, both near the active site (inner cleft: between residues G15 and D157) and the protein surface (outer cleft: between residues E59 and R206) (Supplementary Fig 7B, upper panel). The radius of gyration, a measure of protein compactness, was consequently reduced in ACTC^M305L^ vs. ACTC^WT^ (Supplementary Fig. 7B, lower panel). Moreover, relative to wildtype, both SD2 and SD4 of ACTC^M305L^ showed decreased C_α_ root mean square fluctuations (RMSF) along the most dominant protein motions (Fig. 5c), as well as overall lower flexibilities in cMD simulations (Supplementary Fig. 7C), and along PC1 and PC2 (Supplementary Fig. 7D). Rearrangements of SD1 and SD3 were less pronounced and fluctuated around 1.5 Å and 2 Å for both isoforms. Additional details regarding differences in actin-nucleotide hydrogen bonding can be found in Supplementary Fig. 7E, F.

To further analyze the effect of the M305L mutation on overall structural dynamics of ACTC, and to exclude artifacts from insufficient sampling of the cMD simulations in the actin equilibrium state, we next performed duplicate 500 ns enhanced sampling MD simulations using accelerated MD (aMD) simulations^55–58^. The conformational changes of wildtype and mutant G-actin in the aMD simulations followed a dynamic trend similar to that uncovered by cMD. We computed free energy landscapes (FEL) for both actins along the projections of the first two PCs, i.e. along the largest structural changes in the proteins, by energetic reweighting of the combined aMD simulations using Maclaurin series expansion^59^. The FEL of ACTC^WT^ spanned a wide conformational space along PC1 and PC2 (Fig. 5d and Supplementary Fig. 8A). It was characterized by several minima, indicating a number of low-energy substates of the protein separated by energy barriers of up to ∼2 kcal mol^−1^. In contrast, the FEL of ACTC^M305L^ was more restricted with a single narrow energy basin (Fig. 5e and Supplementary Fig. 8B). Hence, ACTC^M305L^ seems limited to one conformational state, indicative of drastically reduced flexibility (Supplementary Fig. 8C, D).

Previous MD simulations predicted that much of G-actin’s flexibility is retained by F-actin protomers in three different filament models^54^. Therefore, we proceeded to examine the mutation’s effect on intrinsic protein flexibility in a physiologically relevant, filamentous form of ACTC by comparing duplicate 200 ns cMD simulations of fully solvated ACTC^WT^ to ACTC^M305L^ F-actin. The hinge (PC1) and rotational (PC2) motions were also found in F-actin protomers and again represented the dominant structural changes (Supplementary Fig. 9). Consistent with the results of the monomer simulations, the overall protein flexibility, and particularly the RMSFs of SD2 and SD4, were significantly reduced in F-ACTC^M305L^ as compared to wildtype along PC1 and PC2 (Fig. 5f), which suggests the effect of the actin mutation persists post polymerization.

### Assessment of protein allostery and F-actin–Tpm interaction

**Fig. 6:**
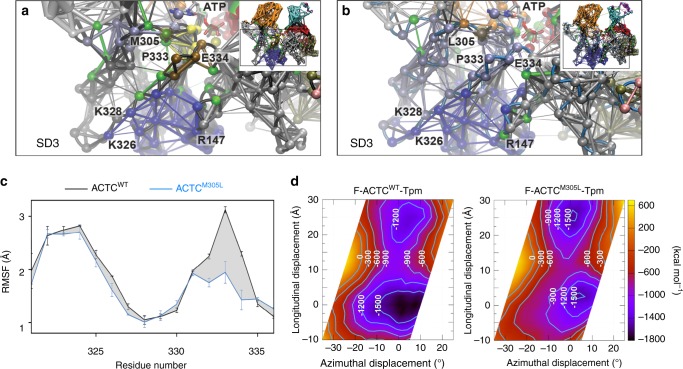
ACTC^M305L^ exhibits altered communication pathways and coupled motions and distorts F-actin–Tpm electrostatic energy landscapes. **a** Dynamical network analysis of cMD simulations of an ACTC^WT^ Tpm-interacting subregion revealed a high degree of coupled motions, represented by the thickness of the connections and weighted according to the correlation data obtained from PCA. Clustered network communities were identified using the Girvan–Newman algorithm and colored individually, indicating regions of concerted movements. Critical residues and connections between two network communities are presented in green. P333 and E334 formed a separate community (brown) that maintained some flexibility and moved independently with respect to K326 and K328. Inset: dynamical network analysis of an entire ACTC^WT^ protomer (see Supplementary Fig. 10A) demarcating the enlarged region discussed above. **b** Dynamical network analysis of cMD simulations of ACTC^M305L^ showed that the coupled motions of the Tpm-interacting subregion around P333 and E334 differed from those of ACTC^WT^ and moved in concert with K326 and K328 and the community surrounding L305 (light blue). Inset: dynamical network analysis of an ACTC^M305L^ protomer (see Supplementary Fig. 10B) demarcating the enlarged region discussed above. **c** Root mean square fluctuations (RMSF) of C_α_ atoms along a stretch of actin residues that forms stable interactions with and facilitates inhibitory positioning of Tpm, measured over the course of enhanced sampling MD simulations. Data are represented as mean ± SEM (*n* = 2). **d** Average electrostatic energy landscapes for wildtype (left) and M305L F-actin–Tpm (right). The origin is set at 0,0, which represents the previously determined energy minimum of the inhibitory, A/B configuration for wildtype F-actin–Tpm^24^, where Tpm is located in a position that would impede myosin binding. The plot is contoured with isolines between −1500 and 0 kcal mol^−1^ in increments of 300 kcal mol^−1^. Note, the broad well around the origin in the wildtype plot is shallower and narrower in the mutant F-actin–Tpm plot. Additionally, a second equivalent energy well is located distal to the A/B site along the mutant filament, which might be predicted to bias Tpm to a non-inhibitory location. Source data are provided as a Source Data file.

Structural changes at distant actin sites can modulate various processes remotely via allosteric communication. Therefore, to further investigate the impact of the M305L substitution, including propagated effects, we performed network analysis of the ACTC MD trajectories (Supplementary Fig. 10A–D). Dynamical network analysis constructs network models by weighting interconnectivity within proteins according to the degree of correlated motions during simulations^60^. As expected, K326 and K328, which function to help confine Tpm to an inhibitory location along the F-actin surface^22,24^, were found within the same network community (dark blue) (Fig. 6a). In wildtype actin this community was interconnected with an adjacent, independent community that centered on P333 and E334 (brown). Note, P333, which projects out from the F-actin backbone, was recently suggested to form a flexible ridge that topologically divides the B- and C-state Tpm positions along thin filaments and may, therefore, represent a crucial determinant of Tpm translocation during regulatory switching^14^. The mutation led to specific changes in the network communities and intercommunity communication (Fig. 6B and Supplementary Fig 10B). For instance, in close proximity to L305, several critical network connections observed in ACTC^WT^ (green connections) were lost, indicating reduced communication in the vicinity of the lesion. The P333- and E334-containing loop no longer moved individually, as found with wildtype actin (Fig. 6a), but was included in the community bearing the mutation (light blue) (Fig. 6b). Furthermore, comparing the isoforms’ allosteric signaling pathways via cross-correlation analysis, revealed altered signal propagation throughout ACTC^M305L^ (Supplementary Fig 10e). Finally, based on the decidedly comparable backbone fluctuations previously noted during MD simulations of wildtype G- and multiple F-actin models^54^, the surface stretch of amino acids containing K326, K328, and E334, which form highly favorable, A/B-state-promoting contacts with Tpm, as well as P333, is expected to exhibit similar and relatively high mobility regardless of actin polymerization status. The M305L substitution however, distinctly reduced flexibility of this subregion (Fig. 6c). The ACTC mutation therefore, appears to decrease overall actin flexibility in the monomeric and filamentous forms, and it disturbs communication between protein subdomains, among regions in the active site, and with residues critical for Tpm interaction and inhibitory positioning along thin filaments.

Given the enhanced contractile and impaired relaxation properties of the mutant fly muscles, the increased motility of M305L actin–Tpm filaments, and the results of our MD simulations, we postulated that the molecular basis of Tpm’s diminished ability to properly inhibit S1 binding could be due to reduced actin flexibility and subregion interconnectivity. Protomer flexibility and precise correlated subregion motion may be required to ensure that the complementary F-actin–Tpm interfacial surfaces and favorable electrostatic interactions persist in order to appropriately confine Tpm to an inhibitory location. Thus, reduced flexibility and intercommunity communication could potentially favor ACTC conformational substates that destabilize the interactions required for A/B-state Tpm positioning along F-actin. The average, composite electrostatic energy map of wildtype F-actin–Tpm, calculated from five individual landscapes using the most predominant ACTC^WT^ conformations extracted from cMD simulations, showed a single, deep, broad energy well that would ideally position Tpm to effectively deter S1 binding (Fig. 6d). In contrast, an electrostatic energy landscape similarly computed for M305L F-actin–Tpm revealed two prominent, discretely located basins. Each well was relatively narrow with roughly equivalent minima that had higher energies compared to wildtype. Inspection of the individual wildtype F-actin–Tpm electrostatic energy landscapes used to create the average landscape, revealed energy wells with minima displaying low positional variation that would consistently place Tpm very close to its typical, energetically optimal, inhibitory A/B-state location (Supplementary Fig. 11A). The M305L F-actin–Tpm energy landscapes however, were characterized by additional new, local minima with a high degree of positional variation (Supplementary Fig. 11B). Therefore, Tpm would not exclusively prefer the A/B-state energy basin and, consequently, may not be constrained to a single, inhibitory location along M305L filaments. Tpm is predicted to exhibit less positional bias and display a larger degree of azimuthal and longitudinal freedom around the M305L F-actin helix, which would result in improper inhibition of actomyosin associations and potentially stimulate human disease.

## Discussion

HCM is characterized by myocardial hypertrophy, impaired relaxation, and hyperdynamic contractile properties^1–3^. The M305L ACTC HCM-causing mutation has been the subject of several in vitro studies, which have yielded both consistent and contradictory results^8–11^. A lack of congruent findings when using only actin, or actin and myosin, calls into question disease mechanisms and suggests higher order regulatory components may be required to resolve HCM pathogenesis, yet the effects of the mutation on thin filaments replete with Tn–Tpm complexes, or on myocyte behavior, have remained untested. Therefore, to help determine potential emergent pathogenic properties, both in vitro and in vivo, we generated transgenic animals that express M305L actin. Our fly models permit integrative analysis of the variant in distinct striated muscles from the molecular through the organ levels. Although the *Drosophila* cardiac tube is relatively simple compared to the mammalian heart, its cellular structure and proteomic makeup are highly conserved and it exhibits pathological responses analogous to those found in higher organisms^31,32,39,40,45,61,62^. Additionally, *Drosophila* IFMs are extremely useful for structural and mechanical analyses of sarcomeric mutations and they provide adequate material for biochemical investigation^31,39,42,44–46^.

When overexpressed in the fly heart, M305L cardiac actin incorporated evenly along the entire length of the thin filaments (Fig. 1). It was not found in discrete subcellular locations nor was it excluded from the sarcomere. Thus, consistent with earlier in vitro studies^9,10^, the amino acid substitution does not appear to impact protein folding or stability in vivo. In the IFMs, even in homozygotes expressing 100% mutant actin, M305L thin filament length was the same as that of controls (Fig. 3b). Therefore, regardless of potential discrepancies in actin polymerization properties or depolymerization rates^9,10^, thin filament formation and integrity are unaffected in mutant sarcomeres and, hence, are unlikely to contribute to altered contractile properties and pathology.

Compared to controls, M305L-expressing heart tubes of three distinct genetic backgrounds, consistently displayed prolonged periods of systolic tension generation (Fig. 2e). Additionally, restricted diastolic volumes and decreased relaxation rates were observed for all mutant genotypes (Fig. 2b–f). The poor relengthening characteristics of M305L cardiomyocytes are indicative of defective relaxation. Diastole is normally characterized by slightly shortened myocytes with modest basal tone^31,39,40,63^ due to a limited number of Ca^2+^-dependent and Ca^2+^-independent actomyosin associations (Fig. 2g). However, comparing the changes in cardiac tube diameters in response to EGTA-EGTA,AM and then to blebbistatin suggested that the markedly shortened cardiomyocytes in latent mutant hearts were not caused by elevated diastolic Ca^2+^ levels, but rather resulted from poor Tpm-dependent blocking, and a disproportionately heightened number of disinhibited, actively cycling myosin cross-bridges (Fig. 2h, i). Irrespective of the actin isoform, the M305L mutation induced IFM hypercontraction, a phenotype previously shown to result from Tpm mispositioning^40,44^ (Figs. 3a and 4b). The destructive syndrome was prevented by genetically ablating myosin (Fig. 4b). This implies that the mutation disrupts the thin filament B-state and permits excessive actomyosin cycling and force production that can be tempered by eliminating 50% of the motors. Finally, heterozygous *Act88F*^*M305L*^ IFM fibers exhibited enhanced Ca^2+^ sensitivity of power development (Fig. 3c), which is indicative of altered contractile regulation as observed with other HCM-causing sarcomeric mutations^4–6^. Therefore, the M305L actin mutation impairs Tpm-mediated inhibition of actomyosin associations and relaxation and promotes muscle force production.

Tpm and F-actin display a structurally synergistic relationship that depends on the complementarity of the surfaces between their three-dimensional forms^19,23^. Tpm’s preshaped contour appears perfectly matched to the relatively flat face of actin subunits along the helical filament^19,21,23^. This structural matching permits a number of optimal interfacial contacts that are fundamental to the Gestalt-binding concept of Tpm regulatory behavior^19,64^. Roughly 30 favorable electrostatic contacts define weak, local F-actin–Tpm interactions^24^. These associations are likely to be essential for permitting the azimuthal, regulatory switching of Tpm, and electrostatic interaction maps reveal they establish an energy basin that biases Tpm to a location where actomyosin cycling is impeded^24^. For example, K326 and K328 of seven successive actin protomers attract oppositely charged residues along Tpm’s entire length^24^. E334 was also among the actin residues purported to make weak, but stabilizing protein-protein contacts with Tpm^24^. These connections, in addition to Tn–F-actin binding, were recently shown to be necessary for contractile inhibition^30,31^.

In accordance with previous findings^54^, our MD simulations revealed that wildtype ACTC, in both the G- and F-actin forms, displays a high degree of flexibility (Fig. 5). Moreover, the wildtype monomer adopted several conformational substates that were separated by low energy barriers. The M305L mutation drastically reduced both actin’s flexibility, especially that of SD2 and 4 as well as the stretch of residues that includes K326, K328, P333, and E334, and the population of conformers (Figs. 5 and 6). Additionally, using network analysis, we discovered that the M305L substitution changes the correlated motions of, and communication between, the protomer’s subregions (Fig. 6). The surface loop containing P333 and E334 lost its ability to function independently, as found in ACTC^WT^, which when combined with diminished flexibility could preclude the formation of certain, important actin conformations and substates required for Tpm-mediated steric gating. Thus, optimal structural matching of the complementary F-actin–Tpm surface contours, and hence Gestalt-binding, may be impaired through disruption of the electrostatic interface, reducing the number of contacts. Weakening interfacial electrostatic associations would predictably deform the normal F-actin–Tpm energy landscape and promote regulatory imbalance. We therefore calculated the average electrostatic energy landscapes (Fig. 6d) from five individual maps, corresponding to the most populated conformational states adopted by wildtype and mutant ACTC during the cMD simulations. The electrostatic energy basin that normally biases Tpm to the inhibitory, A/B-state location along wildtype F-actin was shallower and narrower for the mutant.

Combining the in silico results, the diminished flexibility of P333 suggests an increased kinetic barrier that would slow Tpm M-to-A/B state transitions while the shallower and less discretely localized electrostatic interaction energy basin implies a reduced thermodynamic driving force for the Tpm M-to-A/B state transition, thus enhancing contractile activation and prolonging inactivation. Overall, Tpm would be less confined azimuthally to an inhibitory location, which is consistent with our in vitro motility data that show significantly more mutant filaments are propelled by myosin relative to control (Table 4), and with the inability of mutant fly muscles to completely relax. Additionally, Cassell and Tobacman^65^ indicated that myosin-heads increase the affinity of Tpm for F-actin and Tpm enhances myosin binding to M-state actin, possibly through direct myosin–Tpm interactions^66,67^. Therefore, the M305L mutation could affect the S1 catalytic cycle through effects on F-actin–Tpm–myosin associations, and promote excessive force production and HCM^68^. However, these Tpm–myosin interactions were not tested in our in silico studies, nor do our experimental results, acquired from mutant IFMs, necessarily suggest they are impacted.

Our in vivo, in situ, and in vitro data, generated using numerous cardiac and skeletal muscle fly models, universally show that the M305L actin mutation results in aberrant thin filament-mediated contractile regulation. Mechanistically, our in silico findings imply that in addition to the complementary contours of Tpm and F-actin, a certain degree of actin subunit flexibility and intercommunity communication are required to facilitate the establishment and maintenance of the interfacial electrostatic contacts between the proteins. These contacts, in conjunction with the Tn complex, help establish the thin filament B-state and are vital for proper inhibition of muscle contraction. Therefore, by reducing actin protomer flexibility, the M305L substitution may disrupt F-actin–Tpm interaction and yield excessive force generation due to impaired relaxation, which are the earliest hallmarks of HCM in patients^3^ and the likely cause for myopathy in our animal models.

## Methods

### Construction of transgenes and transgenic *Drosophila*

*UAS-Act57B*^*GFP.WT*^ and -*Act57B*^*WT*^ transgenes, the *Act88F*^*WT*^ genomic construct, and transgenic *Drosophila* were generated as described in Viswanathan et al^30,31^. *UAS-Act57B*^*GFP.M305L*^, *-Act57B*^*M305L*^, and *Act88F*^*M305L*^ transgenes were synthesized by site-directed mutagenesis using the QuikChange mutagenesis kit (Agilent Technologies) and custom primers.

Act57B^M305L^ (+) primer – 5′ GGTGGCACCACCCTGTACCCCGGTATT 3′

Act57B^M305L^ (−) primer – 5′ AATACCGGGGTACAGGGTGGTGCCACC 3′

Act88F^M305L^ (+) primer – 5′ GGCGGTACCACCCTGTACCCTGGTAAG 3′

Act88F^M305L^ (−) primer – 5′ CTTACCAGGGTACAGGGTGGTACCGCC 3′

Transgenic *Drosophila* were created using the PhiC31 integrase system, which ensures each gene integrates at an identical, predetermined genomic location and the results are directly comparable^30,31,69^. Therefore, phenotypic differences in control vs. mutant flies are immediately attributable to the single amino acid substitution in actin.

### *Drosophila* stocks and husbandry

Tissue-specific transgene expression was achieved with the Gal4-UAS expression system^70^. Flies harboring a tissue-restricted Gal4 driver were crossed with flies containing constructs comprised of an UAS followed by an actin transgene^30,31^. The progeny inherit both genes and express the UAS-actin transgene exclusively in the desired muscle tissue. *Hand*^*4.2*^*-Gal4* and *UAS-Stinger* flies were provided by Dr. Rolf Bodmer (Sanford Burnham Prebys Medical Discovery Institute, La Jolla, CA). *4XHand-Gal4* and *Act88F-Gal4* were obtained from Drs. Zhe Han (George Washington University, Washington, D.C.), and Richard Cripps (San Diego State University, San Diego, CA) respectively. The *TinC-Gal4* line was purchased from the Bloomington Stock Center. *Act88F*-null (*ry*^*506*^ *KM88 e*^*s*^) *Drosophila* were obtained from Dr. John Sparrow (University of York, York, UK), and *Mhc*^*10*^ flies from Dr. Sanford Bernstein (San Diego State University, San Diego, CA)^71^. All flies were raised on a standard cornmeal-yeast-sucrose-agar medium at 25 °C unless specified at 29 °C.

For all *Gal4-UAS* crosses, virgin females expressing the GAL4 protein, driven by a tissue-specific promoter, were mated with males carrying the *UAS* transgene. *Act88F-Gal4/Act88F-Gal4;Mhc*^*10*^*/Mhc*^*10*^ stocks were generated by standard mating procedures with balancer chromosomes and were crossed with *UAS-Actin* transgenic lines to generate flies expressing transgenic actin exclusively in the IFM, with a 50% reduction in IFM myosin.

*Act88F* transgenic *Drosophila* had endogenous wildtype (+) actin genes on the third chromosomes, plus an additional (*Act88F*^*WT*^ or *Act88F*^*M305L*^) transgene inserted into the second chromosomes (i.e. *Act88F*^*WT*^/*Act88F*^*WT*^;+*/*+ and *Act88F*^*M305L*^/*Act88F*^*M305L*^;+/+). The transgenes were crossed into the *Act88F*-null *ry*^*506*^ *KM88 e*^*s*^ background by standard mating schemes using balancer chromosomes to generate *Act88F*^*WT*^/*Act88F*^*WT*^;*+*/*KM88 and Act88F*^*M305L*^/*Act88F*^*M305L*^;*+*/*KM88 (*designated as *Act88F*^*WT*^/*Act88F*^*WT*^*;+* and *Act88F*^*M305L*^/*Act88F*^*M305L*^*;+*, respectively). A second cross was performed to establish *Act88F*^*WT*^/*Act88F*^*WT*^;*KM88*/*KM88* and *Act88F*^*M305L*^/*Act88F*^*M305L*^;*KM88*/*KM88* homozygous flies (called *Act88F*^*WT*^/*Act88F*^*WT*^ and *Act88F*^*M305L*^/*Act88F*^*M305L*^, respectively). To generate heterozygotes, with one transgenic and one endogenous *Act88F* gene, *Act88F*^*WT*^/*Act88F*^*WT*^ or *Act88F*^*M305L*^/*Act88F*^*M305L*^ homozygotes were crossed with *w*^*1118*^ yielding *Act88F*^*WT*^;*KM88*/ + and *Act88F*^*M305L*^;*KM88*/ + (referred to as *Act88F*^*WT*^/*+* and *Act88F*^*M305L*^/*+*).

### Confocal microscopy of the *Drosophila* heart

Virgin *Hand-Gal4* female flies were crossed with males carrying the *UAS-Act57B*^*GFP.WT*^ or *UAS-Act57B*^*GFP.M305L*^ transgene to assess the localization of ectopically expressed WT or M305L actin within the progeny’s cardiac myofibrils. GFP-actin-expressing fly hearts were exposed, stained with TRITC-phalloidin, and imaged on a Leica TCS SPE RGBV confocal microscope at ×40 and ×100 magnification as previously detailed^31,72,73^.

### Cardiac physiological analysis

Three-week-old, female *Hand-Gal4* > *, 4XHand-Gal4* > *, or TinC-Gal4* > *UAS-Act57B*^*WT*^ or *UAS-Act57B*^*M305L*^ *Drosophila* hearts were exposed under artificial hemolymph (AH)^31,72^. Cardiac performance was assessed as formerly outlined^37,74^. High-speed videos (~150 fps) of semi-intact beating hearts were recorded with a Hamamatsu Orca-Flash 2.8 digital camera on a Leica DM5000B DIC microscope fitted with a 10×(0.30 N.A.) immersion lens. Cardiac performance was analyzed from the movies using Semi-automated Optical Heartbeat Analysis (SOHA) software^36,37^. M-modes, which provide an edge trace documenting heart wall movement over time, were generated via the program. Myogenic “cardiac output” was calculated as described in Blice-Baum et al^74^.

### Measurement of cardiac dimensions post chemical treatment

Semi-intact hearts of three-week-old *Hand-Gal4* > *UAS-Act57B*^*WT*^ and *Hand-Gal4* > *UAS-Act57B*^*M305L*^ females were imaged and filmed using a 20×(0.50 N.A.) immersion lens^40^. The increase in heart tube diameters following incubation with EGTA-EGTA, AM, to chelate extra- and intracellular Ca^2+^, and then blebbistatin, a small molecule myosin inhibitor, was measured and quantified as previously described^31,39^.

### Flight tests

Flight tests were performed on two-day-old male and female *Drosophila* as described in Drummond et al.^75^. Newly eclosed flies were aged for 2 days at 25 °C, or alternatively at 29 °C where specified. Flies were released into the center of a plexiglass chamber with a light positioned at the top and each assigned a flight index based on direction of flight (6 for upward flight, 4 for horizontal, 2 for downward, or 0 for no flight). The average flight index was calculated by dividing the sum of the individual values by the number of animals tested for each line^75^.

### IFM and myofibril imaging

IFM fluorescent microscopy and myofibril imaging of male and female *Drosophila* were performed as described previously^30,31,76,77^. Briefly, paraformaldehyde fixed, flash frozen thoraces of two-day-old flies were bisected after removing the heads and abdomens. Hemi-thoraces were stained with mouse anti-α-actinin primary and donkey anti-mouse Alexa Fluor 488 secondary antibodies and TRITC-phalloidin, rinsed and imaged on an EVOS^®^ FL cell imaging system (Life Technologies) at ×4 magnification. IFM fibers were carefully cut, removed, and myofibrils gently teased apart and imaged on a Leica TCS SPE RGBV confocal microscope at ×100 magnification. Measurement of thin filament lengths was performed using ImageJ software^30,31,76,77^.

### IFM fiber mechanics

Skinned fibers were prepared from IFMs of 2-3 -day -old female flies as described previously^46^. Briefly, the fiber was mounted on the mechanics apparatus in relaxing solution (pCa 8.0, 12 mM MgATP, 30 mM creatine phosphate, 600 U mL^−1^ creatine phosphokinase, 1 mM free Mg^2+^, 5 mM EGTA, 20 mM N,N-Bis(2-hydroxyethyl)-2-aminoethanesulfonic acid (BES) (pH 7.0), 200 mM ionic strength, adjusted with sodium methanesulfonate, 1 mM dithiothreitol (DTT)) at 15 °C. The fiber was activated at various Ca^2+^ concentrations (pCa 8.0 to 4.0) by partial exchanges with activating solution (same as relaxing solution except pCa 4.0).

Power, elastic modulus, and viscous modulus were measured using sinusoidal analysis as previously outlined^46^. A sinusoidal length change of 0.125% of the muscle length (ML) was applied to the fiber over a 0.5 to 600 Hz frequency range. Power (W m^−3^) was calculated as

1 $$\pi fE_{\rm{v}}\left( {\Delta LL^{ - 1}} \right)^2,$$

where *f* is the frequency of the length perturbation, *E*_*v*_ is the viscous modulus at *f*, and *ΔLL*^−1^ is the amplitude of the length perturbation divided by the muscle length. The power-pCa data and elastic modulus-pCa data were fit using the following equation:

2 $${\mathrm{Power}}\,{\mathrm{or}}\,{\mathrm{Elastic}}\,{\mathrm{modulus}} = A\left( {1 + 10^ \wedge \left( {\left( {pCa_{50} - {\mathrm{pCa}}} \right)\left( h \right)} \right)} \right)^{ - {\mathrm{1}}},$$

where *A* is the maximum power or elastic modulus, pCa_50_ is the inflection point at half maximal power or elastic modulus, and *h* is the slope coefficient. Muscle apparent rate constants *2πb* and *2πc* were determined from plotting and fitting elastic modulus versus viscous modulus as previously described^46,78^.

### IFM protein quantification

Quantitative western blots were performed on IFMs from *Act88F-Gal4*x*yw* and *Act88F-Gal4* > *UAS-Act57B*^*GFP.WT*^ *Drosophila* raised at 25 °C and 29 °C^30^. IFMs dissected from 8 flies were pooled as one biological replicate. Samples were immediately homogenized in Laemmli sample buffer, electrophoresed, and blotted onto a nitrocellulose membrane. Membranes were incubated overnight at 4 °C with primary rabbit anti-actin (Proteintech), rabbit anti-GFP (R&D), and/or goat anti-GAPDH (Genscript) antibodies and were then probed with Donkey anti-rabbit and Donkey anti-goat IRDye secondary antibodies (LI-COR Biosciences) for 60-90 min at room temperature. The membranes were scanned using an Odyssey Infrared Imager (λ = 700 and 800 nm) and analyzed using Odyssey Application Software (v3.030, LI-COR Biosciences). Quantitation was performed on six biological samples with three technical replicates of each. Individual technical replicate values were averaged for each biological replicate. Mean values (±SEM) of transgenic (GFP-labeled) and endogenous actin intensities were determined for the biological replicates and normalized to GAPDH intensities to verify changes in transgenic and endogenous actin abundance due to elevated temperature.

### Actin purification from *Drosophila* IFM

Transgenic IFM actin from *Act88F*^*WT*^/*Act88F*^*WT*^ and *Act88F*^*M305L*^/*Act88F*^*M305L*^ *Drosophila* was purified according to Razzaq, et al.^43^. IFMs from 30-40 flies were pulled and stored in York Modified Glycerol solution overnight at −20 °C. High and low salt buffer extractions selectively isolated monomeric G-actin, which was then polymerized in high salt (to remove tropomyosin) and briefly pelleted at 436,000 x *g* in a Beckman TL-100 ultracentrifuge rotor. Pelleted actin was resolubilized in low salt buffer and mixed in a 1:1 molar ratio with Alexa568-phalloidin and left overnight at 4 °C to equilibrate. 10 nM Alexa568-phalloidin-labeled F-actin was used for in vitro motility experiments.

### In vitro motility

In vitro motility of Act88F^WT^ and Act88F^M305L^ F-actin at 30 °C, in the presence or absence of 300 nM porcine cardiac Tpm, was performed at rabbit psoas myosin concentrations between 12.5 µg ml^−1^ and 100 µg ml^−1^ in the following buffer: 25 mM KCl, 4 mM MgCl_2_, 1 mM EGTA, 25 mM imidazole pH 7.2, 10 mM DTT, 1 mM ATP, 2 mM dextrose, 17 units ml^−1^ glucose oxidase, 125 units ml^−1^ catalase, and 0.5% methyl cellulose. Imaging was conducted on an Olympus IX73 microscope, and TRITC-phalloidin was excited using an X-CITE 120 LED lamp and a 531/40 filter. Emitted light was captured at 593/40 and detected on a Hamamatsu Flash 4LT EMCCD camera. Videos were recorded using HCI imaging software, converted to multipage TIF’s, and imported into ImageJ. Tpm was pre-incubated with TRITC-phalloidin-labeled F-actin prior to flow cell addition. Duplicate assessments from two independent preparations of Act88F^WT^ and Act88F^M305L^ *Drosophila* IFM actin were carried out in parallel. Videos from 4–8 areas of the flow cell were recorded at 1–10 fps for 20 frames total, and filament velocities were measured via automated tracking using the ImageJ plugin, *wrmtrack*^79^. Identical filtering parameters were applied at each condition, i.e. myosin concentration and with or without Tpm, to limit program selection bias. In addition, a very sparse population of labeled F-actin (~1 nM) was used to reduce the number of filament collisions, which oftentimes confounds the tracking algorithm.

At each myosin concentration, and in the presence or absence of Tpm, Act88F^WT^ and Act88F^M305L^ average filament velocities were separately plotted as a function of standard deviation (determined from at least 200 filaments). Data were individually fit to a line (*R*^2^ > ~0.7), and linear regression confirmed that the slopes were statistically equivalent. All data points were then combined and fit to a single line whose *y*-intercept was forced through the origin. The slope of the line represents, on average, the relationship between standard deviation and velocity, which was utilized to establish unique cutoff velocities at each condition tested. Filaments that displaced at an average velocity greater than cutoff were considered movers.

To determine the predicted standard deviation, S.D._pred_, of a population of actin filaments with average velocity *V*_avg_ measured via automated tracking, *V*_avg_ from each condition was divided by the slope. The cutoff velocity *V*_cut_ was then determined by the equation:

3 $${{V}}_{{\rm{cut}}} = {{V}}_{{\rm{avg}}}-0.5 \cdot {\mathrm{S}}{\mathrm{.D}}{{.}}\,_{{{{\rm{pred}}}}}.$$

Thus, a filament was classified as a mover if its average velocity exceeded the average velocity of the population minus one-half of the population’s predicted standard deviation. Percent filaments moving in each area of the flow cell was then determined by dividing the number of movers by filaments counted, and all technical and biological replicate values were averaged together.

### Molecular modeling

Structural models of human α-cardiac actin (ACTC) were generated with MODELLER^80^ using the bovine β-actin crystal structure (PDB ID: 2BTF) as reference. Models of F-actin ACTC^WT^ and ACTC^M305L^ trimers were based on the near-atomic cryo-EM structure of α-skeletal actin (PDB ID: 5JLF). The sequence identities between the ACTC target and those of the template structures are 94% (2BTF) and 99% (5JLF). 20 structures were generated for G- and F-actin, and the MODELLER objective function along with the discrete optimized protein energy (DOPE) score were used for evaluation and selection of the final models. The RMSD values between the final G- and F-actin models and the template structures were 0.148 Å and 0.194 Å, respectively. In all structural models, methylated histidine 73 was generated using the Schrödinger software suite^81^. ATP ∙ Ca^2+^ was placed in the active site of the G-actin models, while ADP ∙ Mg^2+^ was added to each protomer of the F-actin models by superposition with the experimental actin structures. Thus, the reference structures’ nucleotide-protein interaction networks were preserved (Supplementary Fig. 12). For mutant ACTC^M305L^, methionine 305 was replaced with leucine.

### Classical and enhanced sampling MD simulations

A summary of the simulations is presented in Supplementary Table 3. A total of 4 µs simulations was performed with 2 µs enhanced sampling MD simulations. Unless otherwise stated, simulation trajectory analysis was performed using in-house python scripts and the MDAnalysis python package^82,83^, as well as VMD 1.9^84^. All simulations were conducted at the Computer Cluster of the North-German Supercomputing Alliance (HLRN).

Classical molecular dynamics (cMD) simulations were performed with NAMD 2.12^85^ and the CHARMM36 force field^86,87^. The CHARMM General Force Field^88^ was used to obtain parameters of methylated histidine. The structural models of G- or F-actin ACTC^WT^ and ACTC^M305L^ were fully solvated with the TIP3P explicit water model^89^, and charges were neutralized by adding Na^+^ counter ions. A minimum distance of 10 Å between the solutes and the water box edges was used, leading to simulation systems with initial dimensions of ~ 62 × 88 × 92 Å^3^ for G-actin and 147 × 103 × 108 Å^3^ for F-actin. Periodic boundary conditions were applied for all simulations. Langevin dynamics and the Langevin piston method were used to maintain a constant temperature of 310 K and 1 atm pressure. The cutoff for van der Waals interactions and short-range electrostatics was set to 12 Å and the particle-mesh Ewald method^90^ was used for long-range electrostatic interactions. The solvated G- or F-actin systems were initially energy-minimized and equilibrated. Subsequently, production run MD simulations were carried out for 200 ns with an integration time step of 2 fs. cMD simulations of G-actins were performed in triplicates. F-actin cMD simulations were performed in duplicates.

Accelerated molecular dynamics simulations (aMD) significantly enhance the sampling of the conformational space of proteins, with a few hundreds of nanoseconds aMD capturing events on the millisecond-timescale^55^. Therefore, by adding a boost potential to the dihedral angle energy of all individual atoms, in order to lower energy barriers of the protein system, which facilitates the transition between substates, aMD was used to enhance conformational sampling of the actin monomers^56–58^. Parameters for the threshold energy *E* and the acceleration factor *α* were determined from the individual 200 ns cMD simulations of ACTC^WT^ and ACTC^M305L^, and were calculated as

4 $$E = \left\langle {V_{{\rm{dihedral}}}} \right\rangle + 3.5 \cdot N_{{\rm{residues}}},$$

5 $$\alpha = \frac{{3.5}}{5} \cdot N_{{\rm{residues}}},$$

where *N*_residues_ equals the number of protein residues and $V_{{\rm{dihedral}}}$ is the average dihedral energy determined from the cMD simulations. Two independent aMD simulations, for both wildtype and mutant M305L actin, were performed for 500 ns each, starting from the final structures of the 200 ns cMD simulations.

### Principal component analysis

PCA of the individual cMD and aMD simulations was used for dimension reduction of the simulation data and to determine the dominant structural changes and correlated motions in the proteins by diagonalizing the covariance matrix, which was computed for the atomic positional fluctuations of all protein C_α_ atoms. The eigenvectors with the largest eigenvalues—principal components 1 (PC1) and 2 (PC2)—reveal the dominant conformational changes and concerted atomic displacements in the proteins. To visualize the structural changes, PC1 and PC2 of the individual replicas were projected onto the respective average structures of the cMD or aMD simulations. In the case of F-actin trimers, a single protomer (chain B) of ACTC^WT^ or ACTC^M305L^ of the individual replicas was subjected to PCA.

### Computation of G-actin free energy landscapes

Two- and one-dimensional free energy profiles were calculated for PC1 and PC2 by reweighting the two combined aMD simulations of either ACTC^WT^ or ACTC^M305L^ using the PyReweighting toolkit^59^ and Maclaurin series expansion. A bin size of 5 was used for reweighting. PC1 and PC2 were determined through PCA of the first ACTC^WT^ aMD simulation, and all other wildtype and mutant aMDs were projected onto this subspace.

### Dynamical network analysis and cross correlation

Dynamical network communities of highly correlated motions in wildtype and mutant actin monomers were constructed by computing the cross-correlation coefficient for the motion of all C_α_ atom pairs^60^. Network connections (edges) were defined with a cutoff distance of 4.5 Å and occupancy of more than 75% of the simulation time. The betweenness of an edge was calculated as the number of shortest paths that cross that edge. Network connections were weighted according to the cross-correlation data. The Girvan-Newman algorithm^91^ was used to define the network communities of correlated motions. Visualization of the dynamical network communities and the allosteric communication pathways was performed with the NetworkView plugin in VMD^92^.

### Computation of F-actin–Tpm energy landscapes

F-actin–Tpm electrostatic energy landscapes were calculated as outlined previously^21,22^. Briefly, the actin monomer coordinates from the five predominant ACTC^WT^ and ACTC^M305L^ conformations, generated by hierarchical ensemble clustering^93^ of F-actin ACTC cMD simulations, were selected for landscape analysis. The 10 structures were first minimized in Charmm using the images function to include the contacts between monomers present in a filament, in the calculation. Through application of the filament helical symmetry operations, the resulting minimized structures were used to build filaments containing 16 actin monomers. Tpm, at a radius of 43 Å, was then azimuthally rotated in 2.5° increments and longitudinally translated in 2.5 Å increments to ~300 different grid points on the filament surfaces. The origins (0, 0) were set at the previously determined energy minimum of the inhibitory, A/B configuration for wildtype F-actin–Tpm^24^, with positive longitude indicating the pointed end of F-actin. Tpm at each grid point was then docked to these actin models in 0.5 Å increments to a final radius of 39 Å. This docking method was necessary to resolve any poor Tpm–actin contacts that would occur with rotation and translation at the final radius, which are unresolvable through minimization. Conjugate gradient minimization was then performed to an energy gradient of 0.05 kcal mol^−1^ Å^−1^. Electrostatic interaction energies between Tpm and the actin filaments were then calculated for each grid point and the results displayed in Gnuplot 5.2. The values of each grid point for the five wildtype and mutant F-actin–Tpm structures were then averaged and plotted. All plots were contoured with isolines between −1500 and 0 kcal mol^−1^ in increments of 300 kcal mol^−1^.

### Statistical information

All statistical analyses were carried out using GraphPad Prism 7. When measured values were not normally distributed, data were logarithmically transformed and significance assessed via unpaired two-tailed *t*-tests, one-way ANOVAs with Tukey’s multiple comparison tests or two-way ANOVAs with Bonferroni multiple comparison tests as indicated. Repeated measures ANOVAs with Tukey’s multiple comparison tests were performed to study the effects of compounds (EGTA-EGTA,AM and blebbistatin) on the diameters of fly hearts. For flight data, Mann–Whitney tests were used, and for thin filament length measures, Kruskal–Wallis one-way ANOVAs with Dunn’s post hoc tests were employed to assess significance. Large sample sizes, in these instances, assuaged concerns related to heterogeneity in variance. Pooled power:Ca^2+^ data were re-plotted, fit to the Hill equation, and significant differences in fit parameters (pCa_50_, Hill coefficient, maximum power, *f*_max_, and cooperativity) determined via unpaired two-tailed Student’s *t* tests.

### Reporting summary

Further information on research design is available in the Nature Research Reporting Summary linked to this article.

## Supplementary information

Viswanathan et al. Supplementary Information

Description of Additional Supplementary Information

Supplementary Movie 1

Supplementary Movie 2

Supplementary Movie 3

Supplementary Movie 4

Reporting Summary

## Data Availability

Data supporting the findings of this manuscript are available from the corresponding authors upon reasonable request. A reporting summary for this Article is available as a Supplementary Information file. The source data underlying Figs. 2b–i, 3b–c, 4a, 5c, 6c–d, Tables 1–4, supplementary figures 3A–C, 4, 5B–D, 6, 7A–B, E–F, 11 and supplementary tables 1 and 2 are provided in the source data file.

## Source data

Source Data
