# Supplementary material for: A role for actin flexibility in thin filament-mediated contractile regulation and myopathy
Source: Nat Commun. 2020 May 15;11:2417. doi: 10.1038/s41467-020-15922-5 (PMC7229152; doi:10.1038/s41467-020-15922-5)
Supplement: Supplementary file 1 — Viswanathan et al. Supplementary Information [file 41467_2020_15922_MOESM1_ESM.pdf]

Supplementary Information

A role for actin flexibility in thin filament-mediated contractile regulation and myopathy

Viswanathan et al.

Supplementary Methods

Multiple sequence alignment of actins

Amino acid sequence comparison of human and *Drosophila* cardiac and skeletal actins was performed using the Clustal Omega multiple sequence alignment program.

Confocal microscopy of *Drosophila* cardiomyocyte nuclei

Virgin *Hand-Gal4, 4XHand-Gal4,* and *TinC-Gal4* female flies were crossed with males harboring the *UAS-Stinger* transgene, which encodes a nuclear GFP reporter, and reared under distinct experimental conditions (25°C or 29°C) to quantify and assess the strength of each cardiac driver. Nuclei from the undriven *UAS-Stinger* line served as the control to determine potential background expression. To quantitate the abundance of nuclear GFP expressed in the progeny, hearts were exposed, fixed, and mounted on glass slides with ProLong® Gold antifade reagent with DAPI and imaged on a Leica TCS SPE RGBV confocal microscope at 40X magnification. All hearts were imaged in a single day under the same laser and gain/offset settings to control for differences in laser intensity and photobleaching. Fluorescence intensity normalized to nuclear area was quantified using ImageJ software (http://rsb.info.nih.gov/ij/).

Quantitative polymerase chain reaction

Total RNA was isolated from dissected hearts of three-week-old *Hand-Gal4>UAS-Act57B*^{WT} and *Hand-Gal4>UAS-Act57B*^{M305L} female flies using the Quick-RNA microprep kit (Zymo Research Corp.). Reverse transcription to synthesize first strand cDNA was performed using 10ng of total RNA per reaction and the Qiagen QuantiTect Reverse Transcription Kit (Qiagen Inc.). Quantitative polymerase chain reaction was performed on a Bio-Rad CFX96 Real-Time PCR Detection System. Taqman primers (ThermoFisher Scientific) targeting universally-transcribed regions of the L-type Ca^{2+} channel (*Ca-α1D*; Dm01807733), ryanodine receptor (*RyR*; Dm01842311), sarcoplasmic reticulum Ca^{2+}-ATPase (*SERCA*; Dm01820194), Na/Ca exchanger (*Calx*; Dm02136145), inositol-3-phosphate receptor (*Itp-r83A*; Dm02147941) and GAPDH (*Gapdh1*; Dm01843827) were used. Transcript levels were normalized to GAPDH transcript amounts of four biological replicates (15 hearts per biological replicate) with three technical replicates each.

Protein quantification

Quantitative western blots were performed on hearts of *Hand* x *yw, Hand>Act57B*$^{GFP.WT}$ and *Hand>Act57B*$^{GFP.M305L}$ *Drosophila* raised at 25°C and on indirect flight muscles (IFMs) from *Act88F-Gal4* x *yw* and *Act88F-Gal4>UAS-Act57B*$^{GFP.WT}$ raised at 25°C or 29°C. Hearts isolated from 30 flies or IFMs dissected from 8 flies were pooled as one biological replicate. Samples were immediately homogenized in Laemmli sample buffer, electrophoresed, and blotted onto a nitrocellulose membrane. Membranes were incubated overnight at 4°C with primary rabbit anti-actin (Proteintech), rabbit anti-GFP (R&D), and/or goat anti-GAPDH (Genscript) antibodies and were then probed with donkey anti-rabbit and donkey anti-goat IRDye secondary antibodies (LI-COR Biosciences) for 60-90 min at room temperature. The membranes were scanned using an Odyssey Infrared Imager (λ = 700 and 800nm) and analyzed using Odyssey Application Software (v3.030, LI-COR Biosciences). Quantitation in cardiac tissue was performed on eight independent biological samples, with two technical replicates each, and in IFMs on six biological samples with three technical replicates of each. Individual technical replicate values were averaged for each biological replicate. Mean values (±SEM) of transgenic (GFP-labeled) and endogenous actin intensities were determined for the biological replicates to ascertain percent transgenic to total actin in the tissues. For IFM samples, GFP and GAPDH intensities were also determined to verify changes in transgenic and endogenous actin abundance due to elevated temperature (Fig 4A).

Filament counting filters

Under conditions of negligible in vitro F-actin displacement, i.e. at 12.5 μg ml^{-1} myosin concentration, any tracked movement was mostly the result of systematic noise and/or

Brownian motion. This caused the calculation of V_{cut} to be less than the minimum velocity of an active mover, leading to numerous false positives. Under these circumstances, V_{cut} was assigned an average velocity that was indicative of non-myosin based motion (~0.06 μm s^{-1}). Next, for all conditions, a filter was applied that identified moving filaments whose total distance traveled was greater than twice that of net displacement. This sorted filaments into one of two distinct pools whereby movement was either non-directional and non-myosin driven, or it was propelled by myosin but the filaments' starting and ending positions overlapped. For the former, non-specific interactions with the surface were the likely cause for motion, while the latter was probably due to propulsion over a circular bed of myosin. To distinguish between these contrasting motion types, a second, low threshold filter was applied to determine if a filament's maximum velocity, defined as the highest velocity between consecutive frames, was within 1.5 times its average velocity calculated throughout the entire video. If so, filament movement was classified as continuous and likely caused actomyosin cycling. Conversely, if a filament failed the continuous motion test, it was manually inspected and judged accordingly. The low threshold filter rarely produced false positives, and substantially expedited data collection by reducing the number of filaments that required manual inspection.

Supplementary Figures

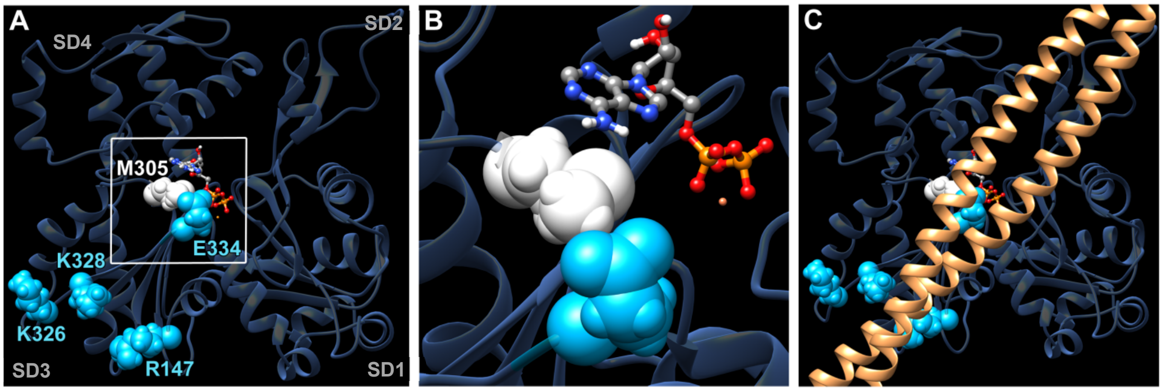

Supplementary Figure 1: Location of the M305L actin HCM mutation.

(A) Location of M305 relative to the actin ATP-binding site (bound ADP shown) and E334, K326, K328, and R147 in subdomain 3 (SD3). K326, K328, and E334 form highly favorable interactions with and help confine Tpm to an inhibitory location along F-actin and thereby assist in striated muscle relaxation. Note M305's proximity to the ATP-binding pocket and E334 (models based on atomic coordinates provided in Orzechowski et al[1]).

(B) Zoomed in view of the inset in (A).

(C) Tpm (brown) superimposed along the surface of an actin monomer. Tpm establishes favorable contacts with colored residues on actin[2].

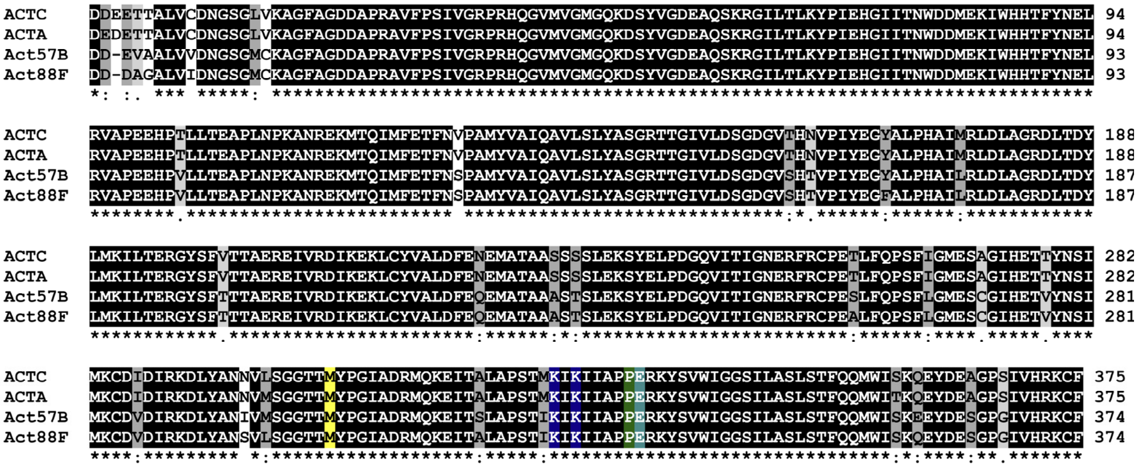

Supplementary Figure 2: Multiple sequence alignment of muscle actins. ACTC - human cardiac muscle actin; ACTA - human skeletal muscle actin; Act57B - *Drosophila* cardiac actin; Act88F - *Drosophila* IFM actin. Residues are shaded based on degree of conservation. An (*) indicates identical amino acids, a (:) indicates residues with high structural similarity, and a (.) indicates residues with low structural similarity. M305 is shaded yellow; K326 and K328 are shaded blue; P333 is shaded green; E334 is shaded cyan, for reference.

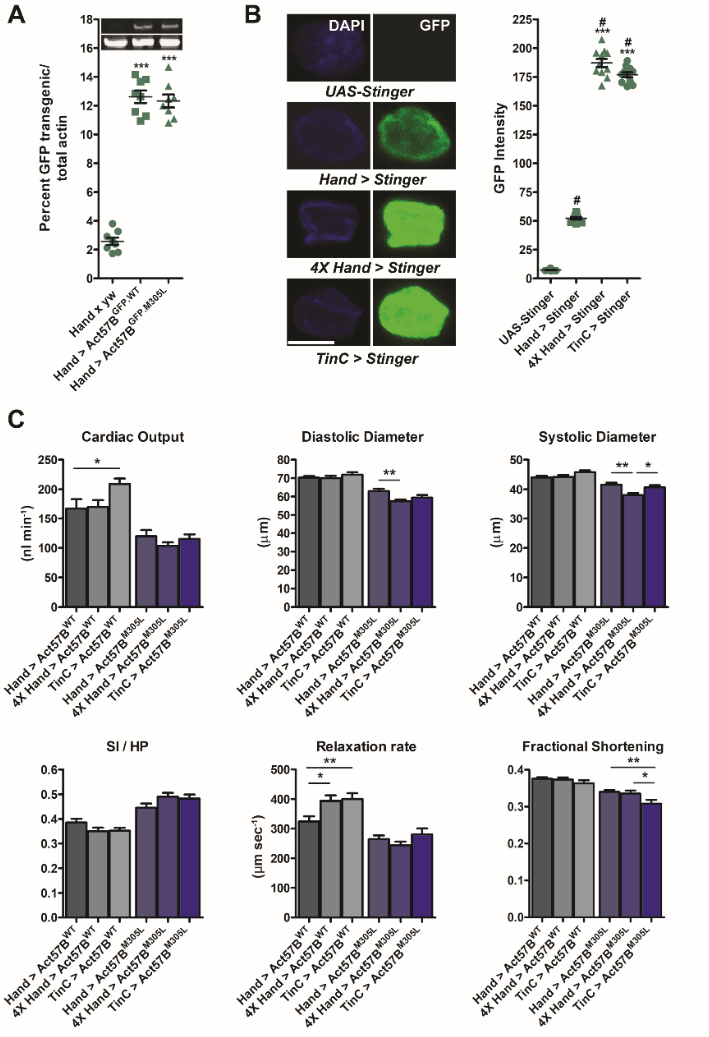

Supplementary Figure 3: M305L actin induces restrictive cardiac physiology independent of the variant's dose.

(A) Quantitative western blot analysis verified that equal amounts of ectopically-expressed Act57B$^{GFP.WT}$ and Act57B$^{GFP.M305L}$ were present in the hearts of three-week-old transgenic flies. Eight individual preparations, each consisting of 30 dissected hearts from *Hand* x *yw* (non-transgenic control), *Hand>Act57B$^{GFP.WT}$*, or *Hand>Act57B$^{GFP.M305L}$* *Drosophila*, were subjected to SDS-PAGE, transferred to nitrocellulose, and probed with rabbit anti-actin and donkey anti-rabbit antibodies. Representative signals from a blot are shown for transgenic Act57B$^{GFP.WT}$ and Act57B$^{GFP.M305L}$ (~70 kDa, top) and endogenous cardiac actin (42 kDa, bottom). The average signal intensities for eight biological replicates revealed that GFP-actin comprised ~10% of total cardiac actin (following subtraction of background signal observed in the non-transgenic control) in both transgenic lines, which was significantly greater (***$P \leq 0.001$) than that determined for control. Significance was assessed via one-way ANOVA with Tukey's multiple comparison test.

(B) Expression levels of transgenes were manipulated by employing multiple cardiac-specific GAL4 driver lines and distinct rearing temperatures (i.e. 25°C or 29°C) (Fig 2B-F). Relative driver strength was determined by crossing *Hand$^{4.2}$-Gal4*, *4XHand-Gal4* at 25°C, or *TinC-GAL4* at 29°C, with flies harboring *UAS-Stinger*, which encodes a nuclear-specific GFP reporter. Nuclear GFP signals were imaged following uniform excitation of the progenies' cardiomyocytes. Quantitation of GFP intensities normalized to nuclei area revealed significantly higher expression of nuclear GFP in all driven lines relative to the undriven *UAS-Stinger* control ($^{\#}P \leq 0.0001$). In addition, roughly four-fold higher transgene expression levels

were noted with the high-dose *4XHand*- and *TinC-Gal4* (29°C) drivers relative to *Hand*$^{4.2}$-*Gal4>UAS-Stinger* line (****P*≤0.001). Significance was assessed via one-way ANOVA with Tukey's multiple comparison test. 12-15 nuclei per heart were imaged from 11 hearts and averaged. Scale bar = 5 μm.

(C) High-dose expression of the mutant actin did not significantly exacerbate cardiac restriction or other pathological phenotypes. Significance was assessed via one-way ANOVAs with Tukey's multiple comparison tests (n = 31-45). **P*≤0.05 and ***P*≤0.01. All data are presented as mean ± SEM. Source data are provided as a Source Data file.

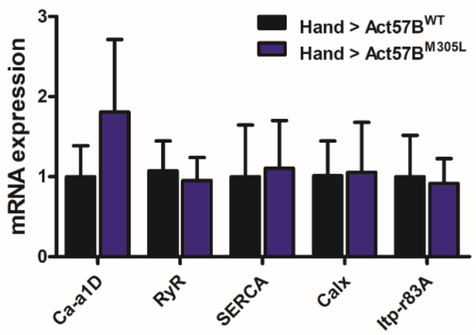

Supplementary Figure 4: M305L actin does not alter Ca^{2+}-handling gene expression. Quantitative polymerase chain reaction measurements of L-type Ca^{2+} channel, ryanodine receptor, sarcoplasmic reticulum Ca^{2+}-ATPase, Na/Ca exchanger, and inositol-3-phosphate receptor transcript levels, in hearts of *Hand-Gal4>UAS-Act57B^{M305L}* relative to *Hand-Gal4>UAS-Act57B^{WT}* control. Two-tailed unpaired t-tests revealed no significant differences in the expression of Ca^{2+}-handling genes between *Hand-Gal4>UAS-Act57B^{WT}* and *Hand-Gal4>UAS-Act57B^{M305L}* hearts. Transcript levels displayed are normalized to GAPDH transcript amounts of four biological replicates (15 hearts per biological replicate) with three technical replicates each. Data are presented as mean ± SEM. Source data are provided as a Source Data file.

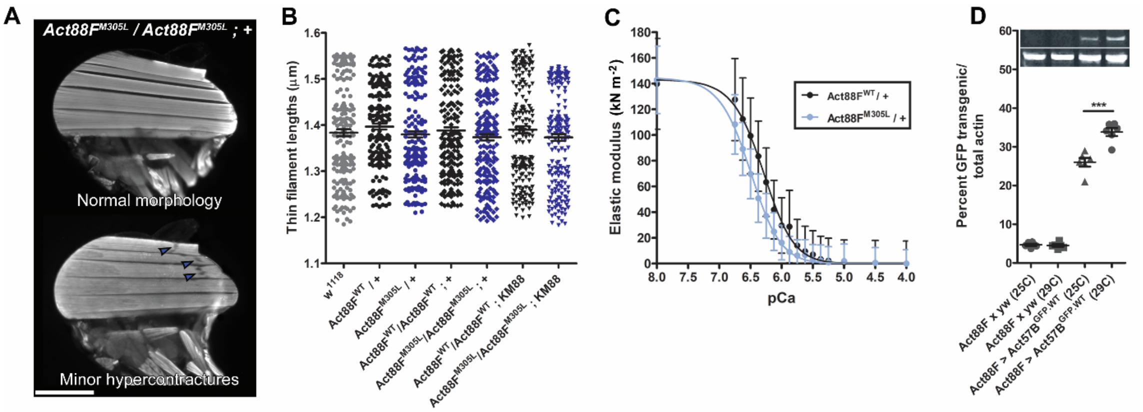

Supplementary Figure 5: M305L actin induces dose-dependent IFM hypercontraction and increased Ca^{2+} sensitivity, but does not alter thin filament lengths.

(A) Elevated doses of M305L actin resulted in hypercontracted IFMs. The majority of *Act88F^{M305L}/Act88F^{M305L}* ;+ flies displayed normal IFM morphology (Fig 3A), yet a small fraction showed minor hypercontractures in a subset of IFMs, the dorsal longitudinal muscles (DLMs) (arrowheads). Scale bar = 250 μm.

(B) IFM thin filament lengths, measured from confocal micrographs (Fig 3B), did not significantly differ among the lines. Significance was assessed via the Kruskal-Wallis one-way ANOVA with Dunn's post-hoc test (n = 200-211).

(C) The elastic modulus-pCa relationship of heterozygous M305L IFM fibers revealed a leftward shift, indicative of increased Ca^{2+} sensitivity. Note that the elastic modulus values were obtained at a muscle length oscillation frequency of 500 Hz, normalized to 0 kN m^{-2} at pCa 4.0 to help visualize changes in Ca^{2+} affinity, and that the values decreased with increasing Ca^{2+} because of the high passive stiffness of relaxed IFM fibers (n = 10). Data points were fit by the Hill equation.

(D) Quantitative western blot analysis confirmed the presence and temperature-induced, elevated abundance of ectopically-expressed Act57B$^{GFP.WT}$ cardiac actin in the IFM. Six individual preparations, each consisting of IFMs dissected from eight *Act88F* x *yw* (non-transgenic control) or *Act88F>Act57B$^{GFP.WT}$ Drosophila* raised at 25°C or 29°C, were subjected to SDS-PAGE, blotted, and probed with rabbit anti-actin and donkey anti-rabbit antibodies. Representative signals from a blot are shown for transgenic Act57B$^{GFP.WT}$ (~70 kDa, top) and endogenous IFM actin (42 kDa, bottom). Average signal intensities revealed that GFP-actin comprised ~20% and ~30% of total IFM actin (following subtraction of background signal observed in controls) in flies reared at 25°C and 29°C, respectively. The amount of transgenic cardiac actin present in the IFMs of flies raised at elevated temperature was significantly greater (***$P \leq 0.001$) than that from flies maintained at 25°C. Significance was assessed via one-way ANOVA with Tukey's multiple comparison test. All data are presented as mean ± SEM. Source data are provided as a Source Data file.

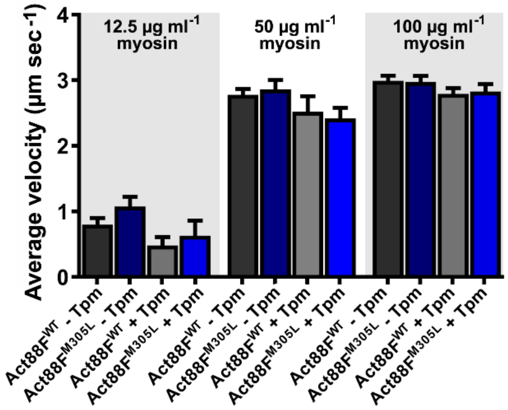

Supplementary Figure 6: Average velocities of Act88F^{M305L} F-actin, with and without Tpm, were similar to Act88F^{WT} F-actin. Average velocities of all filaments classified as movers for each condition (myosin concentration ± Tpm) were weighted according to Viswanathan et al 2017[3] and were indistinguishable under all myosin concentrations and in the presence or absence of Tpm. Significance was assessed using two-way ANOVAs with Bonferroni post-tests (n = 4). All data are presented as mean ± SEM. Source data are provided as a Source Data file.

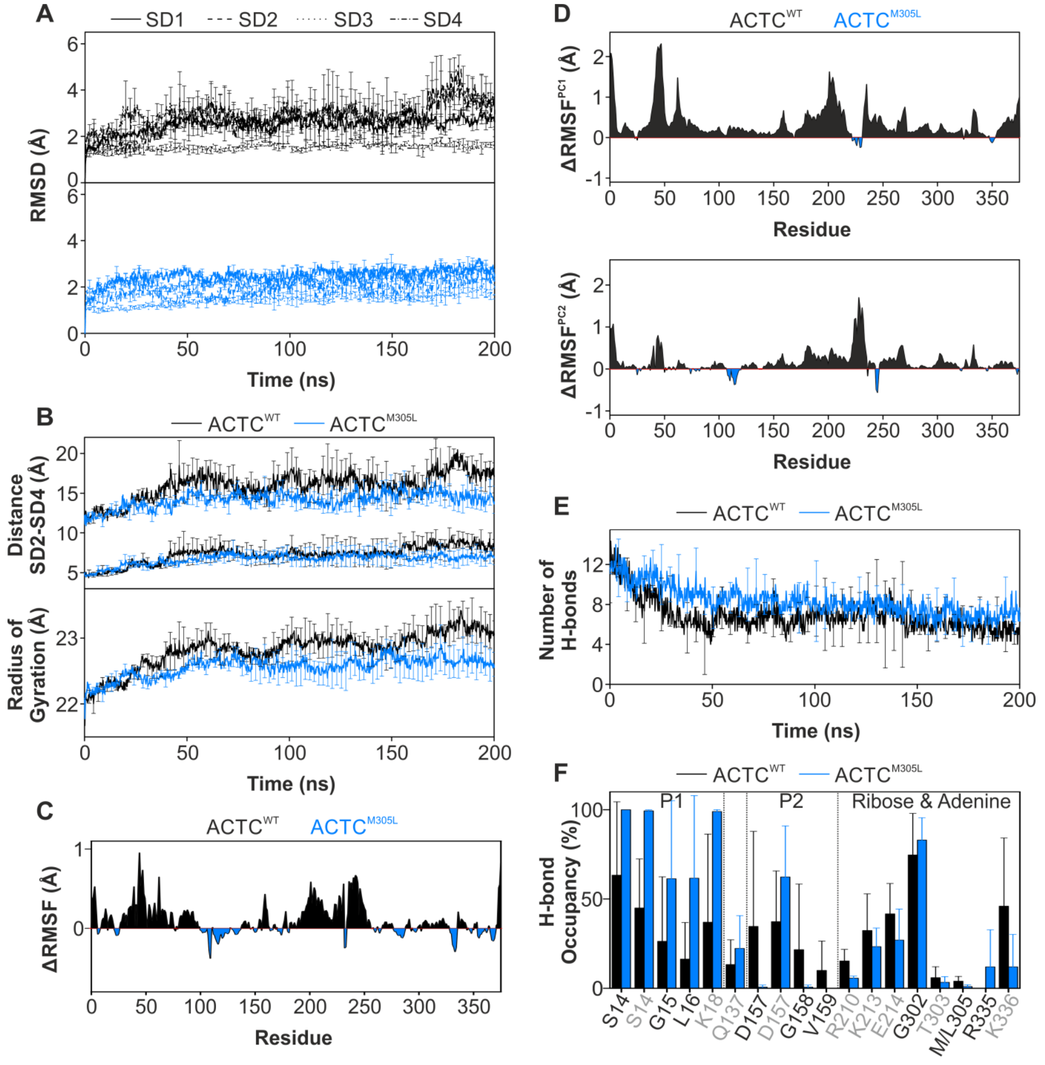

Supplementary Figure 7: Analysis of cMD simulations of ACTC^{WT} and ACTC^{M305L} monomers.

(A) Root mean square deviations (RMSD) of the subdomains (SD) of ACTC^{WT} (black, upper panel) and ACTC^{M305L} (blue, lower panel) over 200 ns cMD simulations, excluding the highly-flexible DNase-binding loop (residues 39 to 50) and the hydrophobic plug (residues 263 to 271).

(B) Time resolved plot of the distances separating SD2 and SD4 (upper panel), monitored between the C_α atoms of residues G15 and D157 (lower curves), as well as E59 and R206 (upper curves) along the cMD trajectories. The protein radius of gyration (lower panel) was monitored over the simulation time (ACTC^{WT}: black, ACTC^{M305L}: blue).

(C) Protein C_α fluctuations relative to their average positions, calculated from the cMD simulations and presented as mean ΔRMSF of ACTC^{WT} – ACTC^{M305L}.

(D) ΔRMSF of ACTC^{WT} – ACTC^{M305L} along PC1 (upper panel). ΔRMSF of ACTC^{WT} – ACTC^{M305L} along PC2 (lower panel).

(E) Time resolved plot of the total number of hydrogen bonds (H-bonds) between ATP and G-actin (ACTC^{WT}: black, ACTC^{M305L}: blue). Concomitant with higher SD movements and remarkably greater flexibility of wildtype G-actin, the total number of hydrogen bonds between the nucleotide ATP and the protein decreased from 12 to five in wildtype, whereas they plateaued at an average of around 7 in the mutant.

(F) Detailed analysis of ATP-interacting residues during the ACTC^{WT} (black) and ACTC^{M305L} (blue) simulations. Values represent percentile occupancies of residue-specific interactions

with either the phosphate-tail (P1 and P2) or the ribose-adenine moiety of ATP, respective of the total number of analyzed frames (backbone interactions: black, side chain interactions: grey). Phosphate binding loop 1 (P1: S14, G15, L16) interactions were particularly more stable in ACTC^{M305L} with occupancies of 60% to 100% of the simulation time for residues S14, G15, L16, and K18 and the ATP phosphate-tail, as well as D157 of phosphate binding loop 2 (P2: D157, G158, V159). A higher number of stable interactions of the nucleotide within the M305L variant might promote the enhanced hydrolysis rates reported previously[4,5]. All data are presented as mean ± SEM. Source data are provided as a Source Data file.

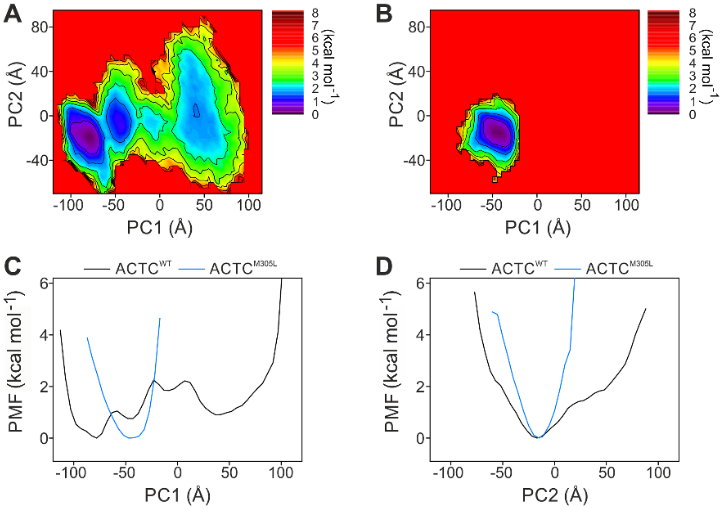

Supplementary Figure 8: Enhanced sampling simulations.

(A) 2D-Free energy profiles of ACTC^{WT} of projections of the two combined 500 ns enhanced sampling MD simulations onto PC1 and PC2. The color bar represents the potential of mean force (PMF) value in kcal mol^{-1}. The free energy landscape (FEL) of ACTC^{WT} spanned a wide conformational space along PC1 and PC2, with ranges of ~200 Å and ~140 Å, respectively.

(B) 2D-Free energy profiles of ACTC^{M305L} of projections of the two combined 500 ns enhanced sampling MD simulations onto PC1 and PC2. The color bar represents the PMF value in kcal mol^{-1}. The FEL of ACTC^{M305L} was restricted, with a narrow energy basin of ~80 Å along PC1 and ~70 Å along PC2.

(C+D) 1D-Free energy profiles of ACTC^{WT} (black) and ACTC^{M305L} (blue) along the first eigenvector (PC1) and the second eigenvector (PC2). ACTC^{M305L} seemed limited to one conformational state around -40 Å (PC1) and -20 Å (PC2), indicative of drastically reduced flexibility.

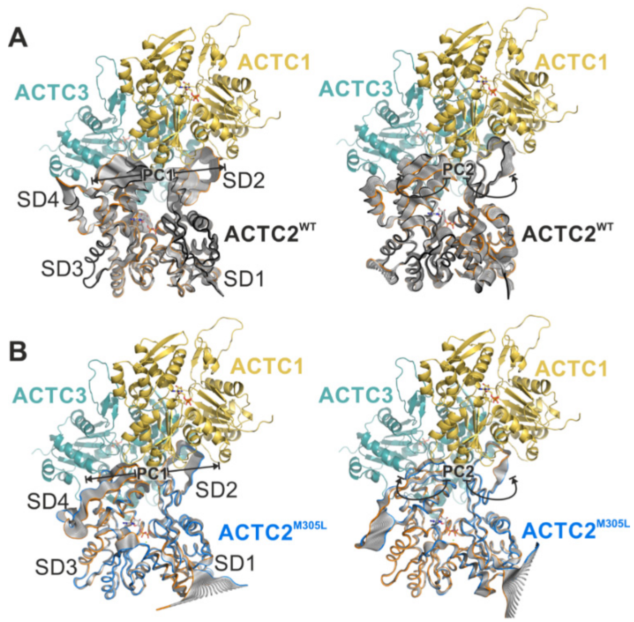

Supplementary Figure 9: Principal component analysis of 200 ns cMD simulations of filamentous ACTC^{WT} and ACTC^{M305L} trimers.

(A) As seen with G-ACTC^{WT} simulations, projection of the first principal component of an ACTC^{WT} protomer showed a similar hinge domain (left panel) and rotational movement (right panel) of SD2 and SD4 in F-actin.

(B) The amplitude of the domain motions in an ACTC^{M305L} protomer was markedly decreased in the filamentous form, in accordance with monomer simulations.

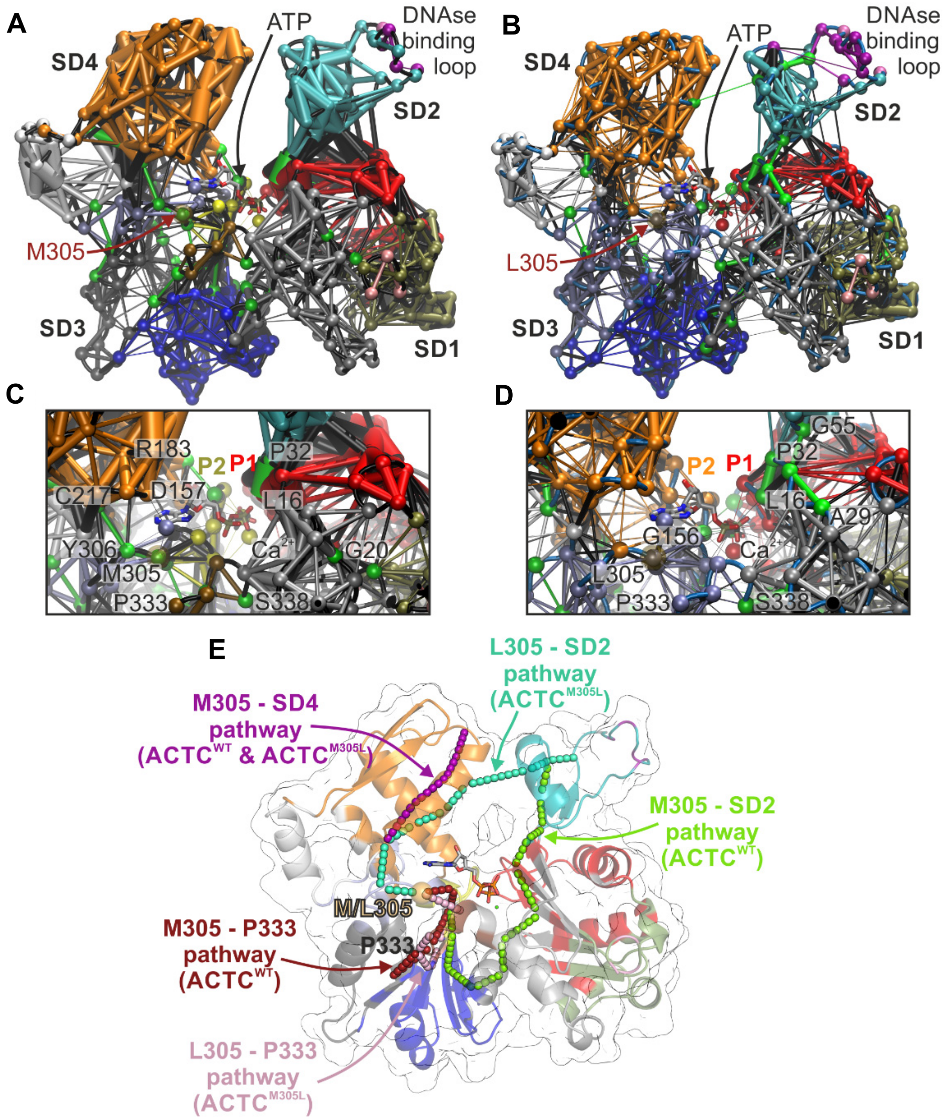

Supplementary Figure 10: Dynamical network analysis of ACTC cMD simulations.
(A) Dynamical network analysis[6] of ACTC^{WT} cMD simulations revealed a high degree of coupled motions, represented by the thickness of the network connections and weighted according to the correlation data obtained from principal component analysis (PCA). Clustered network communities were identified using the Girvan-Newman algorithm and colored individually, indicating regions of concerted movements. Major network communities were formed by residues of SD2 (cyan), and a large substructure of SD4 (orange). Communication among the communities is represented by the green residue nodes and network connections as calculated by their betweeness.
(B) The correlated motions and interconnectivity, overall, in ACTC^{M305L} cMD simulations were drastically reduced as shown by thinner network connections. This correlates with the variant's reduced global flexibility. Network communities also differed in ACTC^{M305L} vs. ACTC^{WT} cMD simulations, with important regions of the actin monomer, including the ATP-binding P2-loop and the Tpm-interacting subregion around P333 and E334, moving in concert with surrounding communities in SD3 and SD4. SD2 and SD4 directly communicated across the ACTC^{M305L} cleft in cMD simulations.
(C) Close-up view of the network communities in ACTC^{WT} around the active site, with ATP bound to phosphate-binding loops P1 (red) and P2 (yellow).

(D) Close-up view of the network communities in ACTC^{M305L} around the active site. In the mutant actin, P2 moved in concert with the major part of the SD4 domain (orange).
(E) Allosteric communication pathways calculated from dynamic network analysis and visualized on the G-actin crystal structure (PDB: 2BTF). The protein is colored according to the communities identified for ACTC^{WT}. Communication between residue 305 and SD2, as well as 305 and P333 was altered in ACTC^{M305L}. Specifically, the communication pathway between L305 and SD2 involved SD4 and may be responsible for the decreased ACTC^{M305L} dynamics (ACTC^{WT} pathways: M305 – P333: dark red, M305 – SD2: green, M305 – SD4: purple; ACTC^{M305L} pathways: L305 – P333: light red, L305 – SD2: cyan, L305 – SD4: purple).

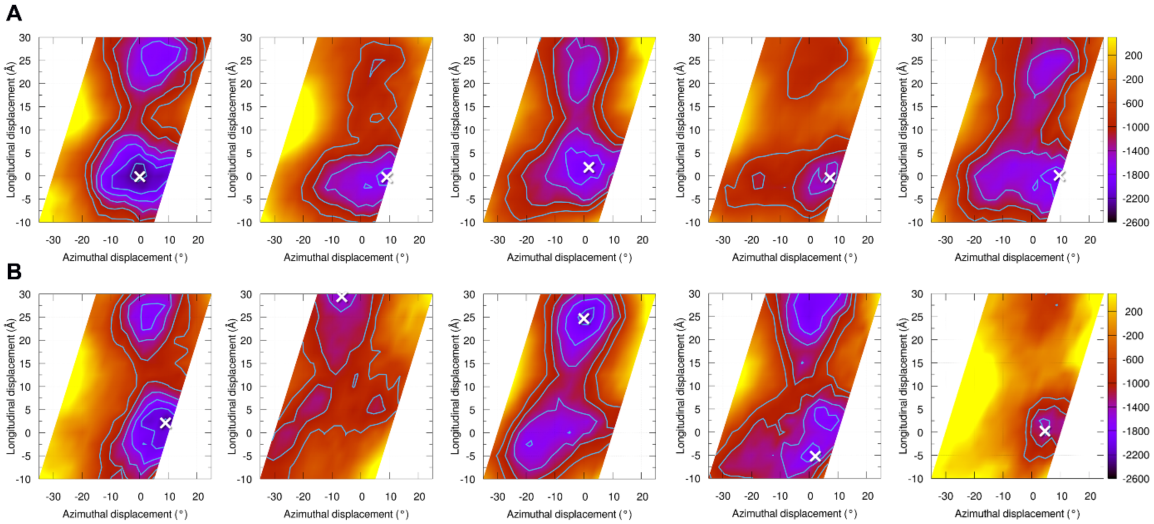

Supplementary Figure 11: F-actin-Tpm electrostatic energy landscapes calculated from the five predominant wildtype and mutant ACTC conformations.

(A) Individual F-ACTC^{WT}-Tpm energy landscapes. Each landscape was computed by creating a filament from the most frequently observed conformations during cMD simulations, as determined by cluster analysis. The five ACTC^{WT} structures were minimized in CHARMM using the images function and actin filaments generated by applying helical symmetry to the minimized monomer (see Methods). Landscape calculations were then performed using our previously described docking methods. In each map the origin is set at 0,0, which represents the energy minimum of the inhibitory, A/B configuration for wildtype F-actin-Tpm[2], where Tpm is located in a position that would impede myosin binding. The plot is contoured with isolines between -1500 and 0 kcal mol^{-1}, in increments of 300 kcal mol^{-1}. The X denotes the position of the energetic minimum for each landscape. Note, all minima for F-ACTC^{WT}-Tpm energy landscapes localized close to the 0,0 origin.

(B) Individual F-ACTC^{M305L}-Tpm energy landscapes. Note the minima (X) for the mutant filaments were not confined to a single region of the landscape that would exclusively bias Tpm to an inhibitory location along F-ACTC^{M305L}.

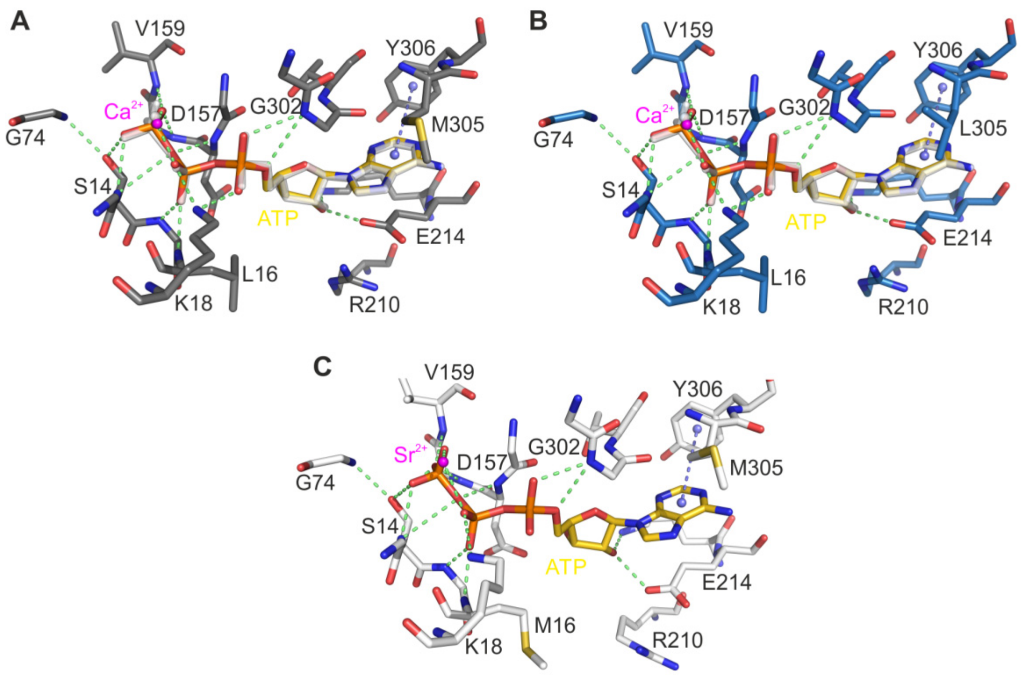

Supplementary Figure 12: Initial positioning of the nucleotide in the wildtype and mutant actin structures.

(A) Close-up view of the nucleotide-binding pocket of $ACTC^{WT}$ (grey) with bound ATP (yellow) and Ca^{2+} (magenta). $ATP \cdot Ca^{2+}$ was placed in the active site according to the reference structure (PDB: 2BTF), maintaining the interaction network with the protein. For comparison, the position of bound $ATP \cdot Sr^{2+}$ in the cytoplasmic β-actin crystal structure (PDB: 2BTF) is shown as transparent white sticks.

(B) Close-up view of the nucleotide-binding pocket of $ACTC^{M305L}$ (blue) with bound ATP (yellow) and Ca^{2+} (magenta).

(C) Close-up view of the nucleotide-binding pocket of the cytoplasmic β-actin crystal structure (PDB: 2BTF) with bound ATP (yellow) and Sr^{2+} (magenta).

Supplementary Tables

Supplementary Table 1: Effects of M305L actin on the *Drosophila* heart. Significant differences between the mutant variant-expressing hearts relative to the appropriate WT control line can be found in Figure 2B-F. Source data are provided as a Source Data file.

	**Cardiac Output ($nl\ min^{-1}$)** Mean ± SEM	**Diastolic Diameter (µm)** Mean ± SEM	**Systolic Diameter (µm)** Mean ± SEM	**Fractional Shortening** Mean ± SEM	**SI/HP** Mean ± SEM	**Relaxation Rate ($\mu m\ sec^{-1}$)** Mean ± SEM
Hand>Act57B^{WT} (n=41)	167.30 ± 15.91	70.33 ± 0.83	43.99 ± 0.56	0.38 ± 0.01	0.38 ± 0.02	324.20 ± 17.48
Hand>Act57B^{M305L} (n=45)	119.70 ± 10.56	63.06 ± 1.17	41.51 ± 0.72	0.34 ± 0.01	0.45 ± 0.02	264.80 ± 12.41
4XHand>Act57B^{WT} (n=33)	170.20 ± 11.55	70.06 ± 1.23	44.16 ± 0.69	0.37 ± 0.01	0.35 ± 0.01	394.00 ± 18.71
4X Hand>Act57B^{M305L} (n=31)	103.20 ± 6.34	57.41 ± 0.93	38.00 ± 0.70	0.34 ± 0.01	0.49 ± 0.02	243.70 ± 12.64
TinC>Act57B^{WT} (n=32)	208.90 ± 9.47	71.89 ± 1.37	45.77 ± 0.67	0.36 ± 0.01	0.35 ± 0.01	400.20 ± 19.77
TinC>Act57B^{M305L} (n=32)	115.00 ± 7.81	59.53 ± 1.35	40.64 ± 0.71	0.31 ± 0.01	0.48 ± 0.02	280.70 ± 19.85

Supplementary Table 2: Elastic modulus (stiffness) measurements show a change in mutant IFM Ca^{2+}sensitivity.

Genotype	pCa_{50}	Hill coefficient	Max. elastic modulus ($W\ m^{-3}$)
Act88F^{WT} / + (n=10)	6.29 ± 0.05	-2.28 ± 0.14	141.20 ± 19.20
Act88F^{M305L} / + (n=10)	6.53 ± 0.04**	-2.20 ± 0.26	143.70 ± 16.30

** Significantly different from *Act88F^{WT}* / + fibers as determined by Student's t-test ($P \leq 0.01$). All data are presented as mean ± SEM. Source data are provided as a Source Data file.

Supplementary Table 3: Overview of performed molecular dynamics simulations.

ID	System	# of protomers	Nucelotide	Simulation Type	Simulation Time (ns)
1	G-Actin wt	1	Ca^{2+}·ATP	cMD	200
2	G-Actin wt	1	Ca^{2+}·ATP	cMD	200
3	G-Actin wt	1	Ca^{2+}·ATP	cMD	200
4	G-Actin wt	1	Ca^{2+}·ATP	dihedral aMD	500
5	G-Actin wt	1	Ca^{2+}·ATP	dihedral aMD	500
6	G-Actin M305L	1	Ca^{2+}·ATP	cMD	200
7	G-Actin M305L	1	Ca^{2+}·ATP	cMD	200
8	G-Actin M305L	1	Ca^{2+}·ATP	cMD	200
9	G-Actin M305L	1	Ca^{2+}·ATP	dihedral aMD	500
10	G-Actin M305L	1	Ca^{2+}·ATP	dihedral aMD	500
11	F-Actin wt (trimer)	3	Mg^{2+}·ADP	cMD	200
12	F-Actin wt (trimer)	3	Mg^{2+}·ADP	cMD	200
13	F-Actin M305L (trimer)	3	Mg^{2+}·ADP	cMD	200
14	F-Actin M305L (trimer)	3	Mg^{2+}·ADP	cMD	200

Supplementary References

1. Orzechowski M, Li XE, Fischer S, Lehman W. An atomic model of the tropomyosin cable on F-actin. *Biophysical journal* **107**, 694-699 (2014).

2. Li XE, Tobacman LS, Mun JY, Craig R, Fischer S, Lehman W. Tropomyosin Position on F-Actin Revealed by Em Reconstruction and Computational Chemistry. *Biophys J* **100**, 1005-1013 (2011).

3. Viswanathan MC*, et al.* Distortion of the Actin A-Triad Results in Contractile Disinhibition and Cardiomyopathy. *Cell Rep* **20**, 2612-2625 (2017).

4. Mundia MM, Demers RW, Chow ML, Perieteanu AA, Dawson JF. Subdomain location of mutations in cardiac actin correlate with type of functional change. *PLoS One* **7**, e36821 (2012). doi: 10.1371/journal.pone.0036821.

5. Müller M*, et al.* Functional characterization of the human alpha-cardiac actin mutations Y166C and M305L involved in hypertrophic cardiomyopathy. *Cell Mol Life Sci* **69**, 3457-3479 (2012).

6. Sethi A, Eargle J, Black AA, Luthey-Schulten Z. Dynamical networks in tRNA: protein complexes. *Proceedings of the National Academy of Sciences* **106**, 6620-6625 (2009).
