# Supplementary material for: A role for actin flexibility in thin filament-mediated contractile regulation and myopathy
Source: Nat Commun. 2020 May 15;11:2417. doi: 10.1038/s41467-020-15922-5 (PMC7229152; doi:10.1038/s41467-020-15922-5)
Supplement: Supplementary file 2 — Description of Additional Supplementary Information [file 41467_2020_15922_MOESM2_ESM.pdf]

Description of Additional Supplementary Files

File Name: Supplementary Movie 1
Description: Projection of PC1 from a representative ACTCWT monomer cMD simulation. PC1 is projected on the average cMD structure and indicates a hinge domain motion of primarily SD2 and SD4.

File Name: Supplementary Movie 2
Description: Projection of PC2 from a representative ACTCWT monomer cMD simulation. PC2 is projected on the average cMD structure and indicates a dominant rotational movement.

File Name: Supplementary Movie 3
Description: Projection of PC1 from a representative ACTCM305L monomer cMD simulation. PC1 is projected on the average cMD structure and shows a decreased hinge domain motion.

File Name: Supplementary Movie 4
Description: Projection of PC2 from a representative ACTCM305L monomer cMD simulation. PC2 is projected on the average cMD structure and illustrates a reduced rotational movement.
